# Repeated sensitization of mice with microfilariae of *Litomosoides sigmodontis* induces pulmonary eosinophilia in an IL-33-dependent manner

**DOI:** 10.1371/journal.ppat.1012071

**Published:** 2024-03-08

**Authors:** Benjamin Lenz, Alexandra Ehrens, Jesuthas Ajendra, Frederic Risch, Joséphine Gal, Anna-Lena Neumann, Julia J. Reichwald, Wiebke Strutz, Henry J. McSorley, Coralie Martin, Achim Hoerauf, Marc P. Hübner

**Affiliations:** 1 Institute for Medical Microbiology, Immunology and Parasitology, University Hospital Bonn, Bonn, Germany; 2 German Center for Infection Research (DZIF), partner site Bonn-Cologne, Bonn, Germany; 3 Unité Molécules de Communication et Adaptation des Microorganismes (MCAM, UMR 7245), Equipe Parasites et Protistes Libres, Muséum National d’Histoire Naturelle, CNRS; CP52, Paris, France; 4 Division of Cell Signaling and Immunology, School of Life Sciences, University of Dundee, Dundee, United Kingdom; Uniformed Services University: Uniformed Services University of the Health Sciences, UNITED STATES

## Abstract

**Background:**

Eosinophilia is a hallmark of helminth infections and eosinophils are essential in the protective immune responses against helminths. Nevertheless, the distinct role of eosinophils during parasitic filarial infection, allergy and autoimmune disease-driven pathology is still not sufficiently understood. In this study, we established a mouse model for microfilariae-induced eosinophilic lung disease (ELD), a manifestation caused by eosinophil hyper-responsiveness within the lung.

**Methods:**

Wild-type (WT) BALB/c mice were sensitized with dead microfilariae (MF) of the rodent filarial nematode *Litomosoides sigmodontis* three times at weekly intervals and subsequently challenged with viable MF to induce ELD. The resulting immune response was compared to non-sensitized WT mice as well as sensitized eosinophil-deficient dblGATA mice using flow cytometry, lung histology and ELISA. Additionally, the impact of IL-33 signaling on ELD development was investigated using the IL-33 antagonist HpARI2.

**Results:**

ELD-induced WT mice displayed an increased type 2 immune response in the lung with increased frequencies of eosinophils, alternatively activated macrophages and group 2 innate lymphoid cells, as well as higher peripheral blood IgE, IL-5 and IL-33 levels in comparison to mice challenged only with viable MF or PBS. ELD mice had an increased MF retention in lung tissue, which was in line with an enhanced MF clearance from peripheral blood. Using eosinophil-deficient dblGATA mice, we demonstrate that eosinophils are essentially involved in driving the type 2 immune response and retention of MF in the lung of ELD mice. Furthermore, we demonstrate that IL-33 drives eosinophil activation *in vitro* and inhibition of IL-33 signaling during ELD induction reduces pulmonary type 2 immune responses, eosinophil activation and alleviates lung lacunarity.

In conclusion, we demonstrate that IL-33 signaling is essentially involved in MF-induced ELD development.

**Summary:**

Our study demonstrates that repeated sensitization of BALB/c mice with *L*. *sigmodontis* MF induces pulmonary eosinophilia in an IL-33-dependent manner. The newly established model recapitulates the characteristic features known to occur during eosinophilic lung diseases (ELD) such as human tropical pulmonary eosinophilia (TPE), which includes the retention of microfilariae in the lung tissue and induction of pulmonary eosinophilia and type 2 immune responses. Our study provides compelling evidence that IL-33 drives eosinophil activation during ELD and that blocking IL-33 signaling using HpARI2 reduces eosinophil activation, eosinophil accumulation in the lung tissue, suppresses type 2 immune responses and mitigates the development of structural damage to the lung. Consequently, IL-33 is a potential therapeutic target to reduce eosinophil-mediated pulmonary pathology.

## Introduction

Pulmonary eosinophilia or eosinophilic lung disease (ELD) can be differentiated into primary (idiopathic) or secondary (extrinsic) causes. Secondary ELD is often a response to allergic processes, medication, drugs, radiation or pathogens, while primary ELD often occurs due to unknown reasons [1]. One of the main causes of secondary ELD are parasite infections [1]. Lymphatic filariasis (elephantiasis) is a neglected tropical disease that is caused by the filarial nematodes *Wuchereria bancrofti*, *Brugia malayi* or *Brugia timori*, which can lead to lymphedema in the extremities as well as the hydrocele in scrotum of men but also manifest as an eosinophilic lung disease called tropical pulmonary eosinophilia (TPE). TPE is a rare but severe complication affecting approximately 1% of lymphatic filariasis patients [2–4]. Symptoms associated with TPE include fever, nocturnal cough, dyspnea, wheezing and pulmonary fibrosis. If left untreated, pulmonary fibrosis can progress and lead to lung failure and eventual death. The disease is classified as a hyper-responsive pulmonary syndrome in response to trapped microfilariae (MF), the filarial progeny, and characterized by a strong infiltration of eosinophils into the lung tissue and increases in parasite-specific and total IgE levels [2–5]. Due to the entrapment of the MF within the lung, TPE patients often lack MF in the peripheral blood. The standard treatment of care is diethylcarbamazine (DEC, 6 mg/kg) given for 21 days, which clears MF and removes pulmonary infiltrates [5,6]. However, 20% of treated patients might relapse within 5 years. Given the dual role of eosinophils in supporting the clearance of MF and mediating lung pathology during TPE, a better understanding of the underlying mechanisms of TPE development is of interest. In 1990, Edwang and Kazura showed that a subcutaneous sensitization of BALB/c mice with *B*. *malayi* MF and a subsequent intravenous challenge with MF induces TPE-like symptoms, including amicrofilaremia in the blood, elevated levels of serum IgE as well as blood and pulmonary eosinophilia [7], thereby mimicking the immune response observed in human TPE patients. Further research in this model by Sharma et al. revealed the development of a type 2 immune response [8] and presence of eosinophils in the lung of TPE mice [9]. Furthermore, they demonstrated a key role for independent phospholipase A2 in the development of lung pathology [10]. In contrast, IL-12 treatment was shown to antagonize TPE development by Mehlotra et al. [11]. Another model to study immune responses during experimental filarial infection is the *Litomosoides sigmodontis* mouse model. This model recapitulates immune responses observed during human filarial infection [12–14], including MF retention in the lung tissue and associated pathology [15–18].

Previously, we demonstrated that eosinophil-deficient dblGATA BALB/c mice are more susceptible to infection with *L*. *sigmodontis*, with all dblGATA mice developing microfilaremia in comparison to 50% of WT BALB/c mice [19]. In addition, WT mice have significantly lower MF numbers compared to dblGATA mice. In accordance, we showed that eosinophils release extracellular DNA traps (ETosis) in response to *L*. *sigmodontis* MF and support their clearance *in vivo* [20]. Similarly, in lymphatic filariasis patients, DEC treatment-induced MF clearance is associated with increased blood eosinophil counts, increased IL-5 serum levels, and is often accompanied by fever, headache and lethargy [21–23]. Mechanisms of MF recognition, MF clearance and MF-driven pathology during filarial infections are to this day insufficiently understood. However, eosinophils are recognized as essential effector cells responding to pathogen- and damage-associated molecular processes during infection.

An important cytokine in the recognition of helminth infection is IL-33. IL-33 is an alarmin predominantly expressed by endothelial and epithelial cells in barrier tissues and released after damage to the respective tissues [24]. ILC2s were found to be the primary recipient of IL-33 signaling after tissue damage via the ST2/IL-1RAcP receptor, releasing high amounts of IL-5 and IL-13, thereby fortifying the eosinophil response. Moreover, eosinophils themselves respond directly to IL-33 [25–31]. Given the importance of type 2 immune responses in protection against filarial infection, it is unsurprising that IL-33 is a potent target of helminth immunomodulation [32]. In line, mice lacking the IL-33 receptor ST2 had an increased microfilaremia due to a reduced MF retention in the spleen, which correlated with decreased eosinophil counts during the infection [33].

In the present study, we introduce an ELD mouse model using the rodent filaria *L*. *sigmodontis* to investigate the role of IL-33 signaling in the development of MF-derived lung inflammation. We demonstrate that repeated sensitization and a challenge of BALB/c WT mice with *L*. *sigmodontis* MF induces pulmonary eosinophilia accompanied by a ELD-like immune response inducing ILC2s, alternatively activated macrophages, highly activated eosinophils and MF retention within the lung. The absence of eosinophils abolishes the formation of a type 2 immune response and inhibiting IL-33 signaling during ELD development reduces pulmonary eosinophilia, ILC2, alternatively activated macrophage and neutrophil frequencies. IL-33 inhibition also results in a reduction in lung lacunarity, indicating a decrease in structural damage within the lung.

## Results

### Microfilariae are rapidly cleared from peripheral blood and retained in the lung tissue of ELD mice in an eosinophil-dependent manner

**Fig 1:**
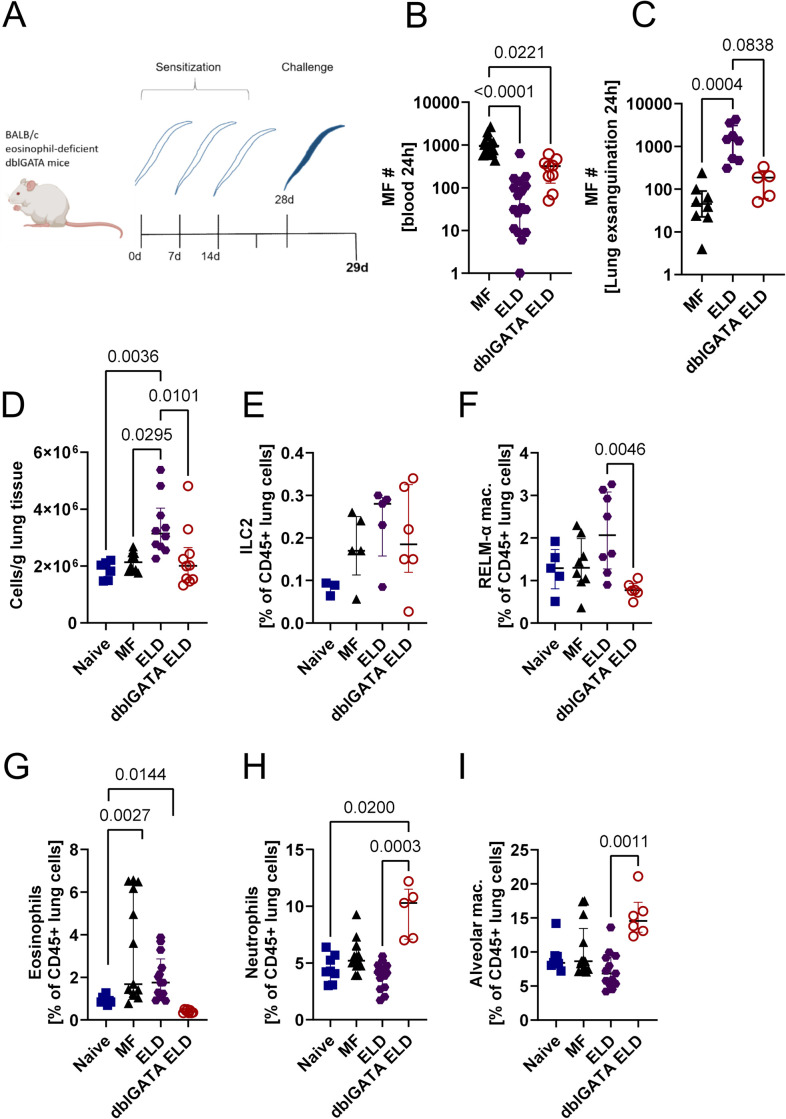
Microfilariae are retained in the lung tissue of ELD mice in an eosinophil-dependent manner. (A) Experimental design (created with BioRender.com). Wild-type ELD and eosinophil-deficient dblGATA ELD mice were sensitized with dead MF and challenged with viable MF two weeks later. Controls received solely the MF challenge (MF) or remained naïve. Analyses were performed one day after MF challenge. (B) Number of microfilariae (MF) in 50 μl of peripheral blood 24 h after challenge injection and (C) exsanguinated from the lung of ELD WT and eosinophil-deficient dblGATA mice as well as MF-only challenged mice (MF). (D) Total cell count/g lung tissue. Cell frequencies of (E) ILC2s (CD45^+^, linage^-^, TCRb^-^, CD90.2^+^, ST2^+^, GATA3^+^), (F) RELM-α positive macrophages (CD45^+^, CD206^+^, Siglec-F^+^, RELM-α^+^), (G) eosinophils (CD45^+^, CD11c^-^, Siglec-F^+^, CD11b^+^), (H) neutrophils (CD45^+^, Ly6G^+^) and (I) alveolar macrophages (CD45^+^, CD206^+^, Siglec-F^+^) in the CD45^+^ lung cell fraction. Data is pooled from 1–3 independent experiments with n = 5–18 animals per group and shown as median with interquartile range. Statistical analysis was performed with Kruskal-Wallis followed by Dunn´s multiple comparison test. p values ≤ 0.05 are shown.

The pathogenesis of ELD in the context of filarial infection is still poorly understood and in order to get a better understanding of the immunological responses involved, we aimed to establish a murine model using the rodent filarial nematode *L*. *sigmodontis*. During human filarial associated ELD such as TPE, MF are retained in the lung tissue resulting in either very low or no detectable MF numbers in peripheral blood [2,4,34]. To verify if induction of ELD using *L*. *sigmodontis* MF leads to a similar MF retention in the lung, BALB/c wild type (WT) mice were sensitized in weekly intervals, a total of three times, with 10^5^ freeze-thawed (dead) *L*. *sigmodontis* MF (Fig 1A). Two weeks after the final sensitization mice were challenged intravenously (i.v.) with viable MF and peripheral blood MF were determined 1 and 24 h after the injection. Retention of MF in the lung was assessed 24 h after the injection. ELD mice had similar peripheral blood MF numbers 1 h after the injection as controls (S1 Fig), but significantly lower numbers 24 h after the injection when compared to non-sensitized MF-challenged mice (Fig 1B). In contrast, ELD mice had significantly more MF in the lung tissue compared to mice receiving only the MF challenge without prior sensitization (Fig 1C), indicating a stronger retention of MF in the lungs of ELD mice. Importantly, ELD induction in eosinophil-deficient dblGATA mice resulted in increased MF numbers in the peripheral blood and lower MF numbers in the lung 24 h after the injection in comparison to WT ELD mice, indicating that eosinophils are involved in the retention of MF in the lung tissue. The retention of MF in the lung tissue of ELD mice was accompanied by an increase in total cell numbers in the lungs of WT ELD mice in comparison to naïve, non-sensitized, MF-only challenged and dblGATA ELD mice (Fig 1D). Type 2 innate lymphoid cell (ILC2s) frequencies were increased by trend 24 h after MF challenge in all groups (Fig 1E), while frequencies of alternatively activated macrophages (AAM, CD206^+^, RELMα^+^) were decreased in dblGATA ELD mice compared to ELD WT mice (Fig 1F). Eosinophil frequencies were significantly increased in both sensitized and non-sensitized animals following MF challenge (Fig 1G). Neutrophil frequencies were highest in dblGATA ELD mice, resulting in a statistically significant difference in comparison to ELD mice 24 h after the MF challenge (Fig 1H). Further, ELD mice and naïve controls showed a comparable frequency of alveolar macrophages (Fig 1I).

**Fig 2:**
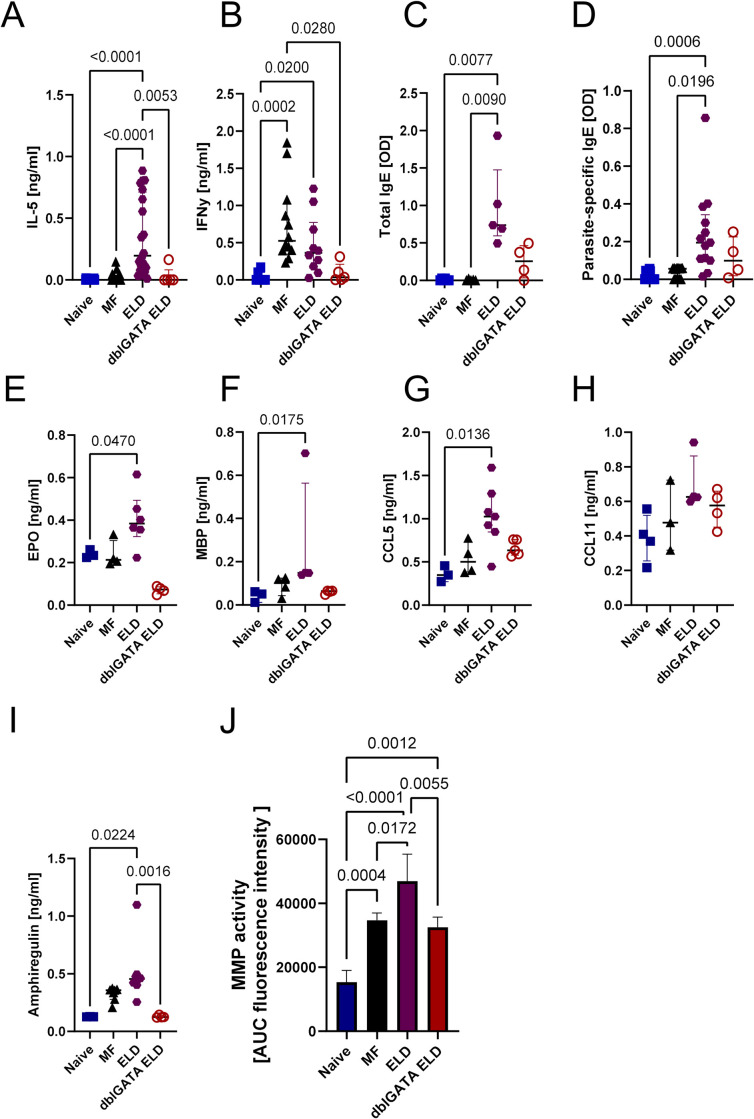
ELD mice have increased levels of eosinophil-associated mediators as well as markers of tissue remodeling activity. Serum (A) IL-5, (B) IFN-y, (C) total IgE and (D) parasite-specific IgE levels from wild-type ELD mice, dblGATA ELD mice, mice challenged with MF (MF) or naïve animals one day after MF challenge. (E) EPO, (F) MBP, (G) CCL5, (H) CCL11 and (I) amphiregulin levels in lung tissue homogenates. (J) MMP activity assay from lung homogenates. Data is pooled from 1–3 independent experiments with n = 4–18 animals per group and shown as median with interquartile range. Statistical analysis was performed with Kruskal-Wallis followed by Dunn´s multiple comparison test. p values ≤ 0.05 are shown.

Complementary, serum IL-5 levels were significantly increased in ELD mice (Fig 2A), but not in mice receiving only the MF challenge. In contrast, IFN-γ levels increased strongest in mice receiving only the MF challenge (Fig 2B) and to a lower extend in ELD mice. Moreover, ELD induction in WT, but not eosinophil-deficient dblGATA mice, led to significantly elevated levels of total and parasite-specific IgE compared to naïve and MF-only challenged animals (Fig 2C and 2D).

In addition, the eosinophil-derived granular proteins eosinophil peroxidase (EPO) and major basic protein (MBP) were significantly elevated in lung homogenates of ELD mice compared to naïve mice (Fig 2E and 2F). Further, the chemokine CCL5 (RANTES) inducing eosinophil migration and activation was significantly increased in ELD mice, while the eosinophil chemotactic chemokine CCL11 (Eotaxin-1) was increased by trend (Fig 2G and 2H). Interestingly, amphiregulin (AREG), a protein associated with tissue repair and mucus production was significantly increased in ELD mice compared to eosinophil deficient ELD dblGATA mice (Fig 2I). To further assess potential matrix remodeling occurring in the lung of ELD mice, we investigated the activity of matrix metalloproteinases (MMPs) in lung homogenates (Fig 2J). MMP activity was significantly increased in ELD, MF challenged and dblGATA ELD mice compared to naïve mice and highest in ELD mice, further indicating increased remodeling activities. Histological analysis of the lung tissue (S2A Fig) did not indicate a significant loss of lung integrity 24 h after challenge.

In summary, 24 h after MF challenge, ELD mice had a retention of MF in the lung tissue, which was dependent on eosinophils and accompanied by increased serum IL-5 as well as total and *L*. *sigmodontis*-specific IgE levels. Further, eosinophil granular proteins (EPO, MBP), eosinophil chemotactic chemokines (CCL5, CCL11) and markers of tissue remodeling (AREG, MMP activity) were increased in ELD mice.

### ELD induction leads to an increased type 2 immune response and eosinophil activation

**Fig 3:**
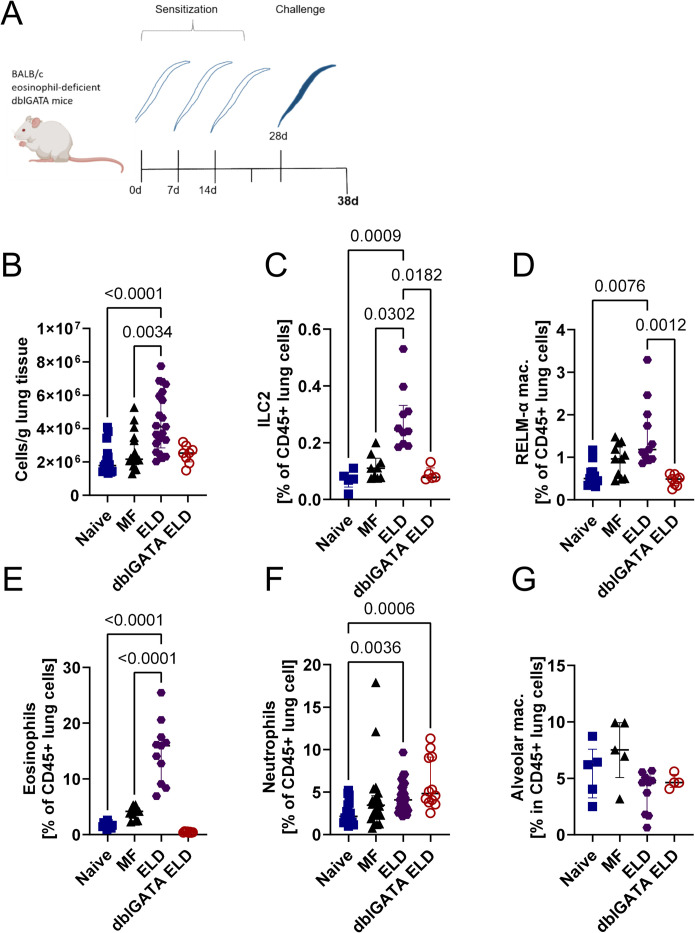
ELD mice display a strong type 2 immune response and eosinophilia in the lung. (A) Experimental design (created with BioRender.com). Wild-type ELD and eosinophil-deficient dblGATA ELD mice were sensitized with dead MF and challenged with viable MF two weeks later. Controls received solely the MF challenge (MF) or remained naïve. Analyses were performed ten days after MF challenge. (B) Total cell count per g of lung tissue for naïve, MF-only challenged, as well as wild-type ELD and dblGATA ELD mice. Frequencies of (C) ILC2s (CD45^+^, linage^-^, TCRb^-^, CD90.2^+^, ST2^+^, GATA3^+^), (D) RELM-α positive macrophages (CD45^+^, CD206^+^, Siglec-F^+^, RELM-α^+^), (E) eosinophils (CD45^+^, CD11c^-^, Siglec-F^+^, CD11b^+^), (F) neutrophils (CD45^+^, Ly6G^+^), and (G) alveolar macrophages (CD45^+^, CD206^+^, Siglec-F^+^) in the CD45^+^ lung cell fraction. Data is pooled from 1–3 independent experiments with n = 6–18 animals per group. Data is shown as median with interquartile range. Statistical analysis was performed with Kruskal-Wallis followed by Dunn´s multiple comparison test. P values ≤ 0.05 are shown.

**Fig 4:**
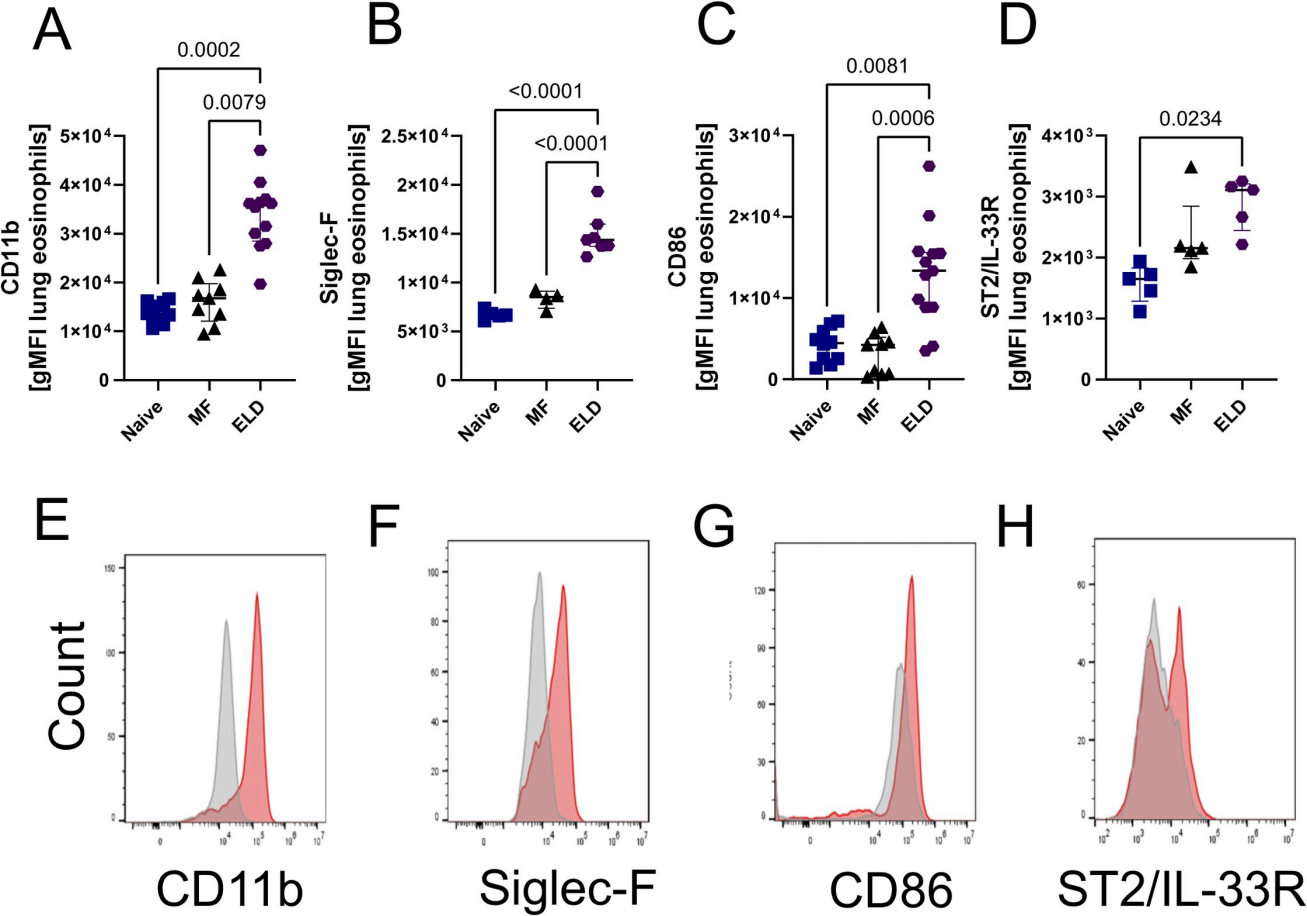
Lung eosinophils are highly activated during ELD. Geometric mean of the fluorescence intensity (gMFI) of (A) CD11b, (B) Siglec-F, (C) CD86 and (D) ST2/IL-33R of lung eosinophils isolated from wild-type ELD mice, mice solely challenged with MF (MF) or naïve animals ten days after MF challenge. (E-H) Representative histograms comparing eosinophils from naïve (grey) and ELD mice (red). (A-D) Data is pooled from 1–3 independent experiments with n = 5–13 mice per group. Data is shown as median with interquartile range. Statistical analysis was performed with Kruskal-Wallis followed by Dunn´s multiple comparison test. p values ≤ 0.05 are shown.

**Fig 5:**
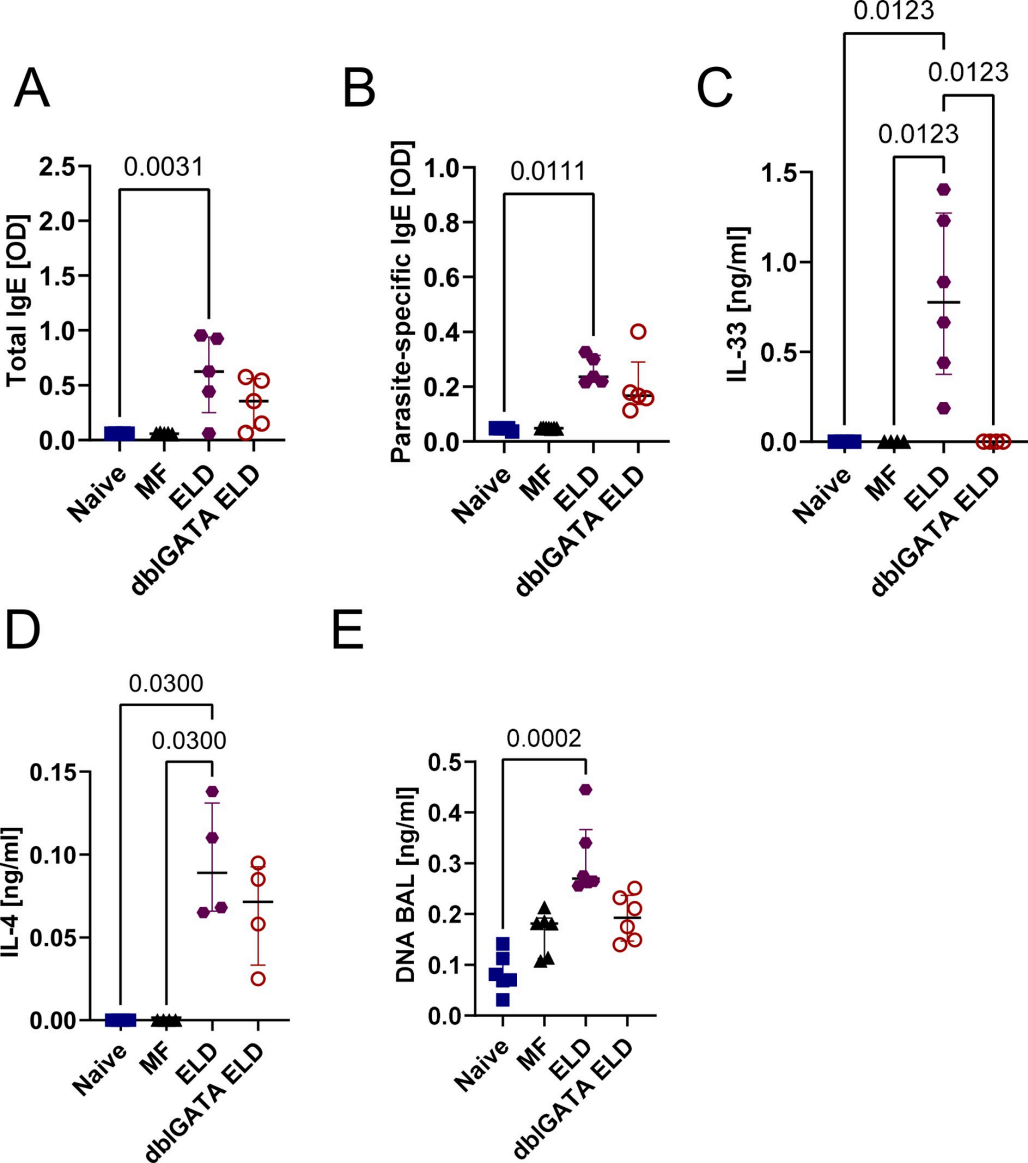
IL-33 serum levels are elevated in ELD mice. Serum levels of (A) total IgE, (B) parasite-specific IgE, (C) IL-33, and (D) IL-4 ten days after the MF challenge in naïve, MF-only challenged, ELD and dblGATA ELD mice. (E) DNA quantification of 20 μl BAL fluid of MF-challenged, ELD and dblGATA ELD mice. One experiment with n = 5–8 mice per group. Data is shown as median with interquartile range. Statistical analysis was performed with Kruskal-Wallis followed by Dunn´s multiple comparison test. p values ≤ 0.05 are shown.

MF were retained in the lung of ELD mice in an eosinophil-dependent manner 24 h after MF challenge. This was accompanied by a shift towards a type 2 immune response with strong eosinophil involvement. To investigate if this type 2 immune response would be maintained without additional MF challenges and potentially induce a pathological process in the lung tissue, we next analyzed the immune response in lung, spleen and broncho-alveolar lavage (BAL) 10 days after ELD induction (Fig 3A). ELD WT mice had a significantly increased cell number in the lung in comparison to naïve and MF-only challenged, non-sensitized controls (Fig 3B). This increase was accompanied by higher frequencies of ILC2s, AAM and eosinophils in the lung tissue, which was not observed in eosinophil-deficient dblGATA mice (Fig 3C–3E). In contrast, lung neutrophil frequencies were increased in both WT and eosinophil-deficient dblGATA ELD mice (Fig 3F). Ten days after the challenge, alveolar macrophages were not significantly altered in ELD, dblGATA ELD or MF challenged mice (Fig 3G). Spleen cell numbers were significantly increased 10 days after MF challenge in both ELD and MF-only challenged, non-sensitized controls in comparison to naïve controls (S3A Fig), while eosinophil frequencies in the spleen were only significantly increased in ELD mice (S3B Fig). Eosinophil frequencies were also significantly increased in the BAL of ELD mice (S3C Fig). Further characterization of lung and BAL eosinophils revealed an elevated expression of CD11b, Siglec-F, CD86 and the IL-33 receptor ST2 in the lung (Fig 4A–4H) and CD11b as well as CD86 for BAL eosinophils (S3D and S3E Fig). Ten days after the MF challenge, total and *L*. *sigmodontis*-specific IgE levels were still increased in the serum of ELD mice (Fig 5A and 5B), while serum IL-5 and IFN-γ were below the detection limit (S4A and S4B Fig) and ELD but not dblGATA ELD mice showed increased serum IL-4 and DNA levels within the BAL compared to naïve mice (Fig 5D and 5E). Interestingly, IL-33 levels within the serum of ELD mice was significantly increased 10 days after MF challenge (Fig 5C). In addition, in some cases, hemorrhages were observed in lungs of MF-challenged, ELD and dblGATA ELD mice (S5A–S5D Fig). ELD mice displayed the highest incidence of hemorrhages with 40.0% (8/20) of WT mice and 37.5% (3/8 p = 0.88 fisher´s exact T-test) of dblGATA ELD mice, while only 20.0% (3/15, p = 0.32 compared to ELD) of MF-challenged and no naïve mice displayed hemorrhages. Taken together, 10 days after MF challenge, ELD mice had significant increases in eosinophil frequencies in lung, BAL as well as spleen and developed a strong type 2 immune response in the lung, which was less pronounced in eosinophil deficient dblGATA mice. The eosinophils were highly activated, which may be mediated by the increased IL-33 levels in ELD mice.

### Eosinophils are activated by IL-33 *in vitro*

**Fig 6:**
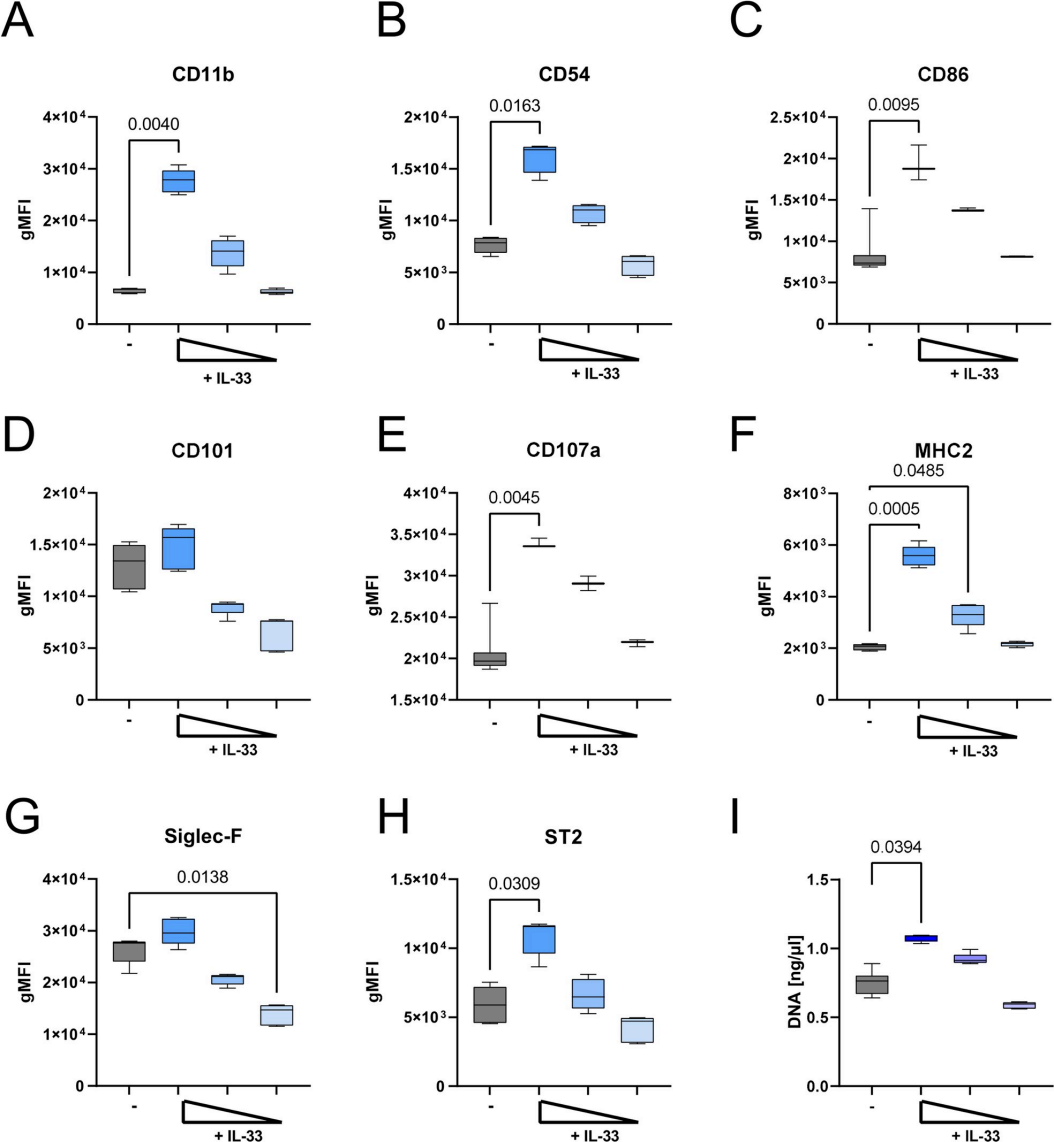
IL-33 activates bone marrow-derived eosinophils in a concentration-dependent manner. Bone marrow-derived eosinophils were stimulated with 0.1, 0.01, 0.001 μg/ml recombinant IL-33 and cultured for 24 h. Geometric mean fluorescence intensity (gMFI) of Siglec-F^+^ eosinophils for (A) CD11b, (B) CD54, (C) CD86, (D) CD101, (E) CD107a, (F) MHC2, (G) Siglec-F and (H) ST2/IL-33R is shown. (I) DNA release of eosinophils after 24 h. Data is pooled from two independent experiments with n = 6–12 per group. Data is shown as median with interquartile range. Statistical analysis was performed with Kruskal-Wallis followed by Dunn´s multiple comparison test. p values ≤ 0.05 are shown.

Eosinophils from ELD mice were highly activated and had an increased expression of the ST2 receptor (Figs 4A–4H and S3D and S3E). Given the strong increase in serum IL-33 levels in ELD mice (Fig 5C), the potential role of IL-33 in driving eosinophil activation was analyzed *in vitro*. Bone marrow-derived eosinophils were co-cultured with 0.1, 0.01 or 0.001 μg/ml IL-33. Eosinophil activation, as indicated by expression of CD11b, CD54, CD86, CD101, CD107a, MHC2, Siglec-F, and ST2, increased in a concentration-dependent manner when IL-33 was added. Eosinophils stimulated with 0.1 μg/ml IL-33 had a significantly increased expression of CD11b, CD54, CD86, CD107a, MHC2, and ST2 in comparison to unstimulated controls (Fig 6A–6H). In line, eosinophils stimulated with 0.1 μg/ml IL-33 released significantly more DNA compared to unstimulated eosinophils (Fig 6I).

### IL-33 signaling is essential for ELD-induced type 2 immune responses

**Fig 7:**
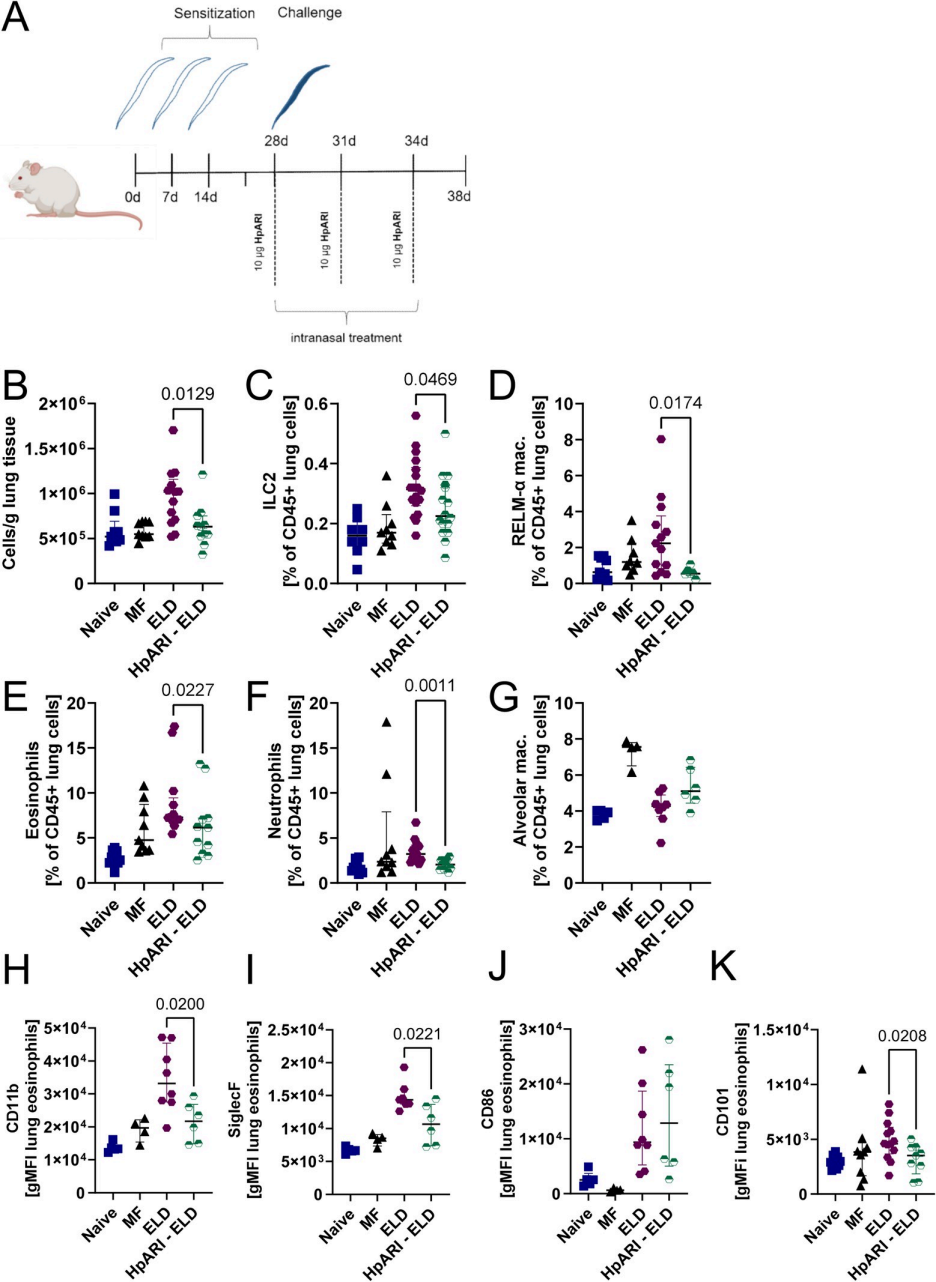
IL-33 signaling is essential for ELD-induced type 2 immune responses. (A) Experimental design (created with BioRender.com). Wild-type ELD mice were sensitized with dead MF and challenged with viable MF two weeks later. A subset of ELD animals received HpARI2 to block IL-33 signaling. Controls received solely the MF challenge (MF) or remained naïve. Analyses were performed ten days after MF challenge. (B) Total lung cell count. Frequencies of (C) ILC2s (CD45^+^, linage^-^, TCRb^-^, CD90.2^+^, ST2^+^, GATA3^+^), (D) RELM-α positive macrophages (CD45^+^, CD206^+^, Siglec-F^+^, RELM-α^+^), (E) eosinophils (CD45^+^, CD11c^-^, Siglec-F^+^, CD11b^+^), (F) neutrophils (CD45^+^, Ly6G^+^), and (G) alveolar macrophages (CD45^+^, CD206^+^, Siglec-F^+^). (H-K) Geometric mean fluorescence intensity (gMFI) of (H) CD11b, (I) Siglec-F, (J) CD86, and (K) CD101 of lung eosinophils in naïve, MF-challenged, ELD and HpARI2-treated ELD mice. Data is pooled from two independent experiments with n = 6–15 animals per group. Data is shown as median with interquartile range. Statistical analysis was performed with Mann-Whitney-U test between ELD and HpARI2 ELD with p values ≤ 0.05 shown.

Considering that eosinophils are essential for ELD induction and IL-33 levels are significantly increased in ELD mice, but not dblGATA ELD mice, which trigger eosinophil activation *in vitro*, we next evaluated if IL-33 is important for the development of MF-associated ELD. Therefore, IL-33 signaling was blocked using the IL-33 suppressor *Heligmosomoides polygyrus* Alarmin Release Inhibitor 2 (HpARI2) [32]. HpARI2 was administered intranasally every three days starting 1 h before and ending ten days post challenge with viable MF (Fig 7A). ELD mice that received HpARI2 had a significantly decreased total lung cell count compared to untreated ELD animals (Fig 7B). In addition, frequencies of ILC2s, RELMα-positive macrophages, eosinophils and neutrophils were lower than in ELD animals not treated with HpARI2 (Figs 7C–7F and S6A and S6B). Similar to the aforementioned experiments, alveolar macrophage frequencies were not significantly altered in ELD mice (Fig 7G). Total and parasite-specific IgE levels were comparable between ELD and HpARI2-treated ELD mice (S7A and S7B Fig). Reduction of eosinophils, ILC2s, RELMα-positive macrophages and neutrophils in HpARI2-treated mice indicates that IL-33 drives ELD-associated immune responses. Accordingly, eosinophils contained in the lung tissue had a significantly lower expression of CD11b, Siglec-F, CD101, but not CD86, when animals were treated with HpARI2 in comparison to untreated ELD mice, indicating a decreased activation (Fig 7H–7K).

### Eosinophil-deficiency and inhibiting IL-33 signaling reduce matrix remodeling and lacunarity

**Fig 8:**
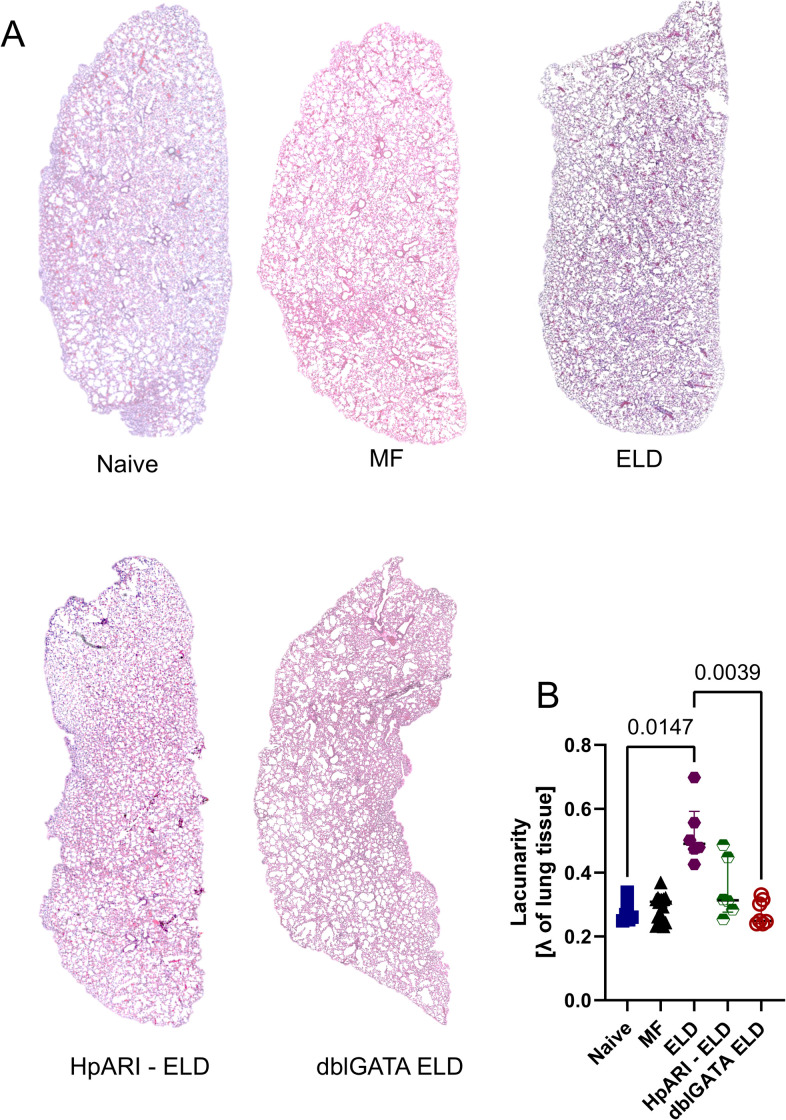
Eosinophil deficiency and HpARI2 treatment reduce ELD-induced lung lacunarity. (A) Representative lung tissue sections of naïve, MF-challenged (MF), ELD, dblGATA ELD and HpARI2-treated ELD mice (ELD-HpARI) stained with hematoxylin and eosin ten days after MF challenge. (B) Lacunarity of 5 μm lung tissue sections. Lacunarity was assessed in 50 randomly generated lung regions of interest of naïve, MF-challenged, ELD, dblGATA ELD and HpARI2-treated ELD animals and pooled, representing one data point per mouse. n = 3–5 mice, two sections per mouse, one out of two representative experiments are shown. Statistical analysis was performed with Kruskal-Wallis followed by Dunn´s multiple comparison test. p values ≤ 0.05 are shown.

**Fig 9:**
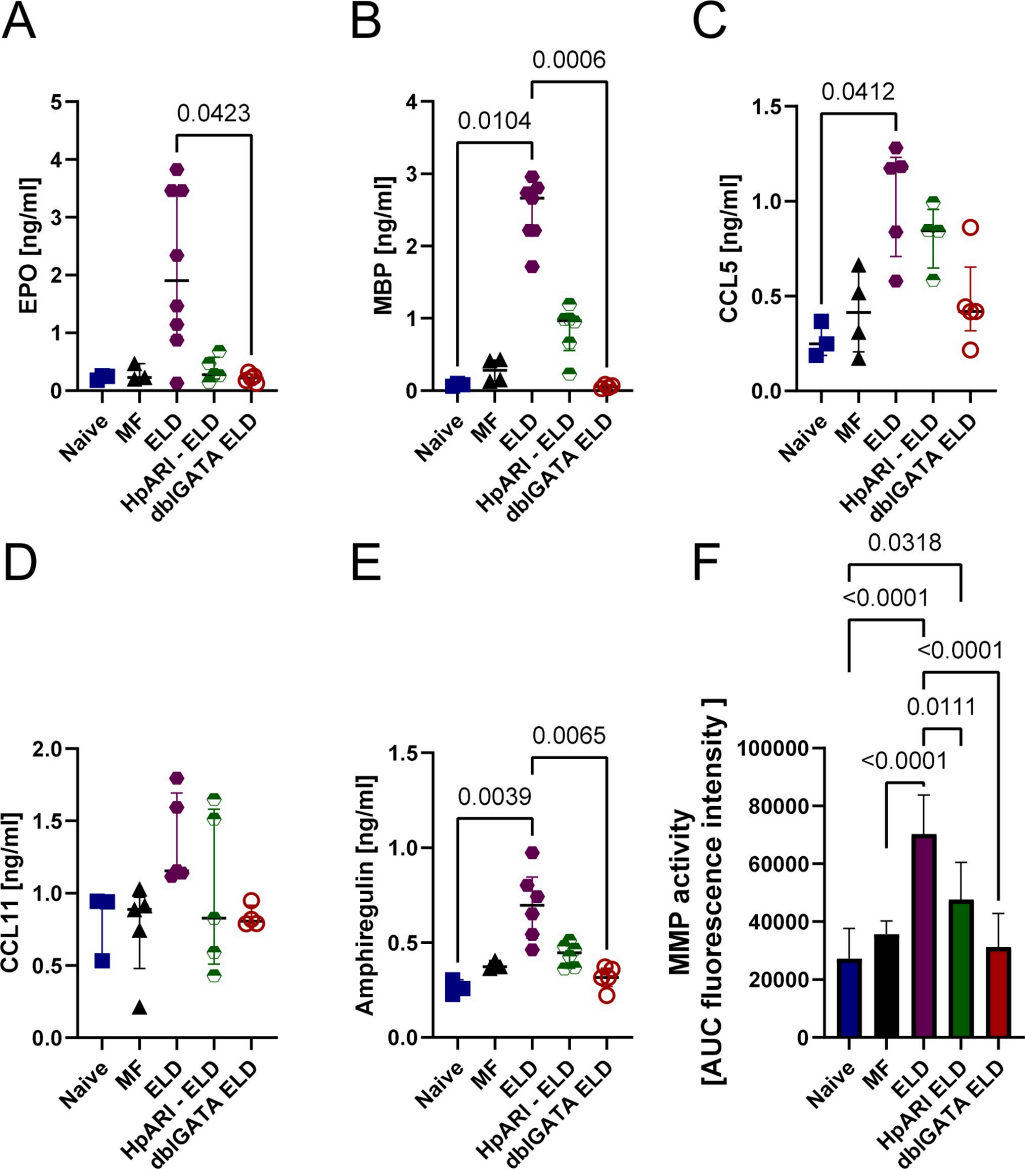
Tissue remodeling and eosinophil-associated granular proteins and chemokines are reduced after HpARI2 treatment. A) EPO, (B) MBP, (C) CCL5, (D) CCL11 and (E) amphiregulin levels in lung tissue homogenates ten days after MF challenge. (F) MMP activity assay from lung homogenates. Data shown is from one experiment with n = 4–9 animals per group and presented as median with interquartile range. Statistical analysis was performed with Kruskal-Wallis followed by Dunn´s multiple comparison test. p values ≤ 0.05 are shown.

Finally, histological analysis of the lung lacunarity, i.e., disintegration of the lung structure, was compared between ELD, HpARI2-treated ELD and dblGATA ELD animals (Fig 8A). The lacunarity was significantly increased in ELD mice but not dblGATA ELD mice, indicating damage to the lung integrity was in part mediated by eosinophils (Fig 8B). Importantly, lacunarity was also reduced after HpARI2 treatment (Fig 8A and 8B). In line, treatment of HpARI2 strongly reduced EPO and MPB from lung homogenates (Fig 9A and 9B). However, HpARI2 treatment and absence of eosinophils did not significantly reduce the lung protein content of the eosinophil chemoattractants CCL5 and CCL11 (Fig 9C and 9D). Finally, AREG and MMP activity were reduced when eosinophils were absent as well as in HpARI2 treated mice, indicating decreased tissue repair and remodeling processes (Fig 9E and 9F).

Our findings highlight that the activation of eosinophils, the initiation of type 2 immune responses, and the resulting lung tissue damage observed in ELD mice were ameliorated through the inhibition of IL-33 signaling. Further, absence of eosinophils hampers the formation of a type 2 immune response and decreases markers of tissue remodeling.

## Discussion

In our current study, we have successfully established a mouse model for MF-associated eosinophilic lung disease by utilizing the rodent filarial nematode *L*. *sigmodontis*. Through this model, we demonstrated the crucial involvement of eosinophils and IL-33 in the formation of ELD-associated immune responses. The *L*. *sigmodontis* ELD mouse model recapitulated key features observed in human ELD, namely the induction of type 2 immune responses as characterized by increased frequencies of AAMs and eosinophils, increased IL-5 as well as total IgE levels. This is further in line with previous observations from human TPE patients where eosinophilia (12-fold increase of eosinophils in the lower respiratory tract) was accompanied by increased IL-5 and parasite-specific IgE levels, eosinophil degranulation and retention of MF in the lung tissue [35,36]. The results obtained in the *L*. *sigmodontis* ELD mouse model also reflect previous findings made in a TPE mouse model using *B*. *malayi* MF, where eosinophils, AAM, IL-5 as well as IgE levels were increased [8]. Our study provides compelling evidence that eosinophils are essential for the MF retention in the lung. This was evident as the immunological changes observed in ELD mice were reduced in eosinophil-deficient dblGATA mice. In contrast, dblGATA ELD mice had, by frequency, increases in neutrophils and alveolar macrophages. This was likely caused by the absence of eosinophils shifting the total percentages of other immune cells in the lung tissue. Accordingly, absolute numbers of neutrophils and alveolar macrophages between ELD and dblGATA ELD mice were statistically not different (S7C and S7D Fig).

Our study provides further evidence that repeated sensitization with MF induces TPE-like immune responses. In comparison to *B*. *malayi* MF, the size of *L*. *sigmodontis* MF is substantially smaller. *B*. *malayi* MF are approximately 260 x 8 μm in size, while *L*. *sigmodontis* MF are approximately one third of the size with 78–85 x 3.5 μm [37,38]. This may have implications for the outcome of both models, as the size of the MF may impact the immune responses and the clogging of capillaries. Given that human microvessels are double the size compared to mice [39], the *L*. *sigmodontis* ELD mouse model may be more reflective for human TPE-associated responses even though a non-TPE-inducing filarial species was used. However, future studies should assess disease development and progression in the presence of the immunomodulatory adult worms to identify possible underlying mechanisms of TPE development. The *L*. *sigmodontis* ELD mouse model offers the possibility to investigate this during chronic adult worm infections. This will be of importance, as the adult worms may interfere with different pathways, including the IL-33 singling and eosinophil activation.

Here, our *in vitro* experiments demonstrate that IL-33 activates bone marrow-derived eosinophils in a concentration-dependent manner, whereas Siglec-F expression and CD101 expression decreased below the values of the controls. Thus, lower concentrations of IL-33 may have a different effect on eosinophils compared to high concentrations, i.e., an inhibitory effect, which has been observed for other cytokines like IL-1 and IL-6 [40,41]. That said, the exact impact IL-33 signaling may have on eosinophils during ELD should be further investigated *in vivo* during chronic filarial infection.

Eosinophils from ELD mice were highly activated in an IL-33-dependent manner. Given that we observed an increase in pulmonary ILC2s in ELD mice, we speculate that ILC2s support the development of pulmonary inflammation by orchestrating the recruitment of eosinophils and exacerbating disease progression in a similar manner as it was described in papain-induced airway inflammation. Here, ILC2s expand in response to IL-33 and release IL-5 together with Th2 cells [42,43], triggering eosinophil maturation and IL-33 receptor expression [44–47]. Accordingly, blocking IL-33 signaling in our ELD model reduced ILC2 frequencies, eosinophil frequencies as well as eosinophil activation. Due to the lung centered nature of the study, dose and rout of administration were adapted from the original HpARI2 publication [32]. The type 2 immune response formed in our ELD model was significantly reduced but not completely abolished by the intranasal HpARI2 treatment. An even stronger effect may be achieved by a systemic blocking of IL-33 signaling by either injecting HpARI2 intravenously or using IL-33 deficient mice.

Future studies could further define the distinct role of eosinophils and their subsets in recognizing MF and ELD/TPE pathology. On one hand, eosinophils are associated with protective immune responses against MF during filarial infections of mice and humans [19–21,48–50], on the other hand, eosinophils can cause airway hyper-responsiveness during allergies and asthma or cause tissue damage [51]. During MF-associated ELD in mice, eosinophils seem to promote both, clearance of MF from the peripheral blood by retaining MF in the lung tissue and diminishing lung integrity. Eosinophils from ELD mice of this study were highly activated in an IL-33-dependent manner as was shown by their increased expression of Siglec-F, CD11b, CD86, ST2, and CD101. Such a role of IL-33 to drive eosinophilia, facilitating eosinophil adhesion and activation of eosinophils was previously described, further supporting our findings [31,52,53]. Furthermore, subsets of eosinophils were recently categorized as regulatory eosinophils (CD101^low^ Siglec-F^int.^) and inflammatory eosinophils (CD101^high^ Siglec-F^high^), the latter being increased in the sputum of asthma patients [54]. Similarly, Gang et al. recently demonstrated that a similar increase in inflammatory eosinophils occurs in the TPE *Brugia spp*. model [55], which could indicate that these eosinophils might be associated with lung hyper-responsiveness, MF clearance and potentially lung pathology. Accordingly, tissue repair mechanisms, i.e., release of AREG and enhanced matrix metalloprotease activity in ELD mice of our study suggest that the loss of lung tissue integrity could be caused by a prolonged repair mechanism and fibrosis which was dependent on eosinophils and IL-33 signaling.

Interestingly, our *in vitro* experiments indicate that IL-33 stimulation triggers also extracellular DNA trap formation (ETosis) from eosinophils (Fig 6I), which was previously shown by our group to trap MF and supports MF removal [20]. Whether extracellular DNA traps, increased cell influx and cell-antibody-MF interactions are responsible for the MF-induced pathology by further tightening the small blood vessels surrounding the alveoli [20,56] is still unclear and should be analyzed in future studies.

Taken together, we postulate a role of IL-33 signaling during the development of MF-induced pulmonary eosinophilia, as inhibition of IL-33 signaling during ELD development reduced eosinophil frequency and activation, associated type 2 immune responses and lung tissue damage.

## Material and methods

### Ethics statement

Experiments were performed with 6–8 week old female BALB/c WT or dblGATA knock-out mice. WT mice were obtained from Janvier (Saint-Berthevin, France) and kept in individually ventilated cages within the animal facility of the Institute for Medical Microbiology, Immunology and Parasitology (IMMIP). BALB/c dblGATA mice were bred at the Haus für Experimentelle Therapie, University Hospital Bonn. Animal experiments were performed according to the EU Directive 2010/63/EU and were approved by the state authorities (AZ 81–02.04.2020.A103, Landesamt für Natur-, Umwelt- und Verbraucherschutz, Recklinghausen, Germany). Water and food were provided *ad libitum*. Animals were checked daily for wellbeing. Animals were weighed once a week during the experiments to additionally monitor health conditions.

### Microfilariae purification

MF were purified as described previously [20]. In short, cotton rats (*Sigmodon hispidus)* naturally infected with *L*. *sigmodontis* were bled into EDTA tubes (Sarstedt AG & Co. KG, Nümbrecht, Germany). The blood was diluted 1:2 in pre-warmed RPMI-1640 media (Gibco, ThermoFisher Scientific, California, USA) and layered on top of a 25% and 30% sucrose gradient (Carl Roth, Karlsruhe, Germany) of 0.25 M iso-osmotic percoll (Merck, Darmstadt, Germany). The gradient was centrifuged at 300g for 30 min without breaks. The white layer containing the MF was collected and washed 3 times with pre-warmed RPMI-1640. MF were counted in a Neubauer chamber (Laboroptik GmbH, Friedrichsdorf, Germany) and adjusted to 1x 10^5^ in 150 μl PBS (Dulbecco´s PBS, Gibco, ThermoFisher Scientific, California, USA). The MF were checked for viability via microscopy.

### Bone marrow-derived eosinophil culture

Bone marrow was isolated from hind legs of WT BALB/c mice using a 20-gauge needle and 5 ml RPMI-1640 for each bone. Isolated cells were centrifuged (400g, 10 min, 4°C) and red blood cells were lysed by adding 1 ml of red blood cell lysis buffer (eBioscience, ThermoFisher Scientific, California, USA) for 5 min at room temperature (RT). Cells were washed with 9 ml PBS (Gibco, ThermoFisher Scientific, California, USA) and centrifuged at 400g at RT for 10 min, adjusted to 1x 10^6^/ml in RPMI-1640 with the addition of 20% fetal calf serum, penicillin (10000 U/ml), streptomycin (100 mg/ml), glutaMAX, gentamycin (50 mg/ml) (Gibco, ThermoFisher Scientific, California, USA for all contents). Cells were cultured with 100 ng/ml FMS-like tyrosine kinase 3 ligand (FLT3L) and 100 ng/ml stem cell factor (SCF) (PeproTech Inc., ThermoFisher Scientific, California, USA) for 4 days and subsequently with 20 ng/ml IL-5 (PeproTech Inc., ThermoFisher Scientific, California, USA) until day 12. Half of the medium was replaced on day 2, 6, 10 and the whole medium was replaced on day 4 and 8. Cells were adjusted using CASY automated cell counter (OLS OMNI Life Science GmbH & Co. KG, Bremen, Germany). Cells were centrifuged and adjusted to 1x 10^6^/ml in the aforementioned culture media with 10% fetal calf serum. Cell purity was determined using flow cytometry and cell viability was assessed using the Annexin-V-PI staining kit (ThermoFisher Scientiffic, California, USA) and was above 80% purity and 55–85% viability (S8A and S8B Fig).

### Sensitization and induction of ELD mice

The sensitization protocol was adjusted from Egwang and Kazura, 1990 [7]. Mice were injected subcutaneously into the neck fold with 1x 10^5^ dead (killed by freezing) MF in 100 μl PBS once a week for three weeks. Two weeks later, mice were injected with 1x 10^5^ freshly isolated, living motile MF in 150 μl PBS (Dulbecco´s PBS, Gibco, ThermoFisher Scientific, California, USA) intravenously. Control mice received 150 μl PBS.

### HpARI2 treatment of ELD mice

Full length HpARI2 with a 6-His tag was expressed in Expi293 cells, and purified by nickel affinity chromatography, as previously described [57,58]. HpARI2 was used to inhibit IL-33 signaling during ELD development. Therefore, 10 μg HpARI2 in 10 μl PBS (Gibco, ThermoFisher Scientific, California, USA) was administered intranasally under anesthesia induced with 2% isoflurane (Piramal Critical Care, New-Delhi, India). For ELD induction, mice were sensitized as usual once a week for three weeks total with dead MF and two weeks later, starting 1 h before the final MF challenge, mice were treated every three days with HpARI2. Control mice received 10 μl PBS intranasally.

### Broncho-alveolar lavage

After euthanasia of the animals, the thorax was opened carefully to allow the lungs to dilate completely. The throat of the animals was cut open to expose the trachea. A 20G Vasofix (B. Braun Melsungen, Melsungen, Germany) intravenous vein catheter was inserted into the trachea and the lung was flushed with 1 ml PBS. The first ml was centrifuged at 400g for 10 min at 4°C and the supernatant was used for the analysis of cytokines. The lung was flushed with additional 4 ml PBS (Gibco, ThermoFisher Scientific, California, USA). Finally, 1 ml of PBS was injected into the lung to dilate the lobes before paraffin (Carl Roth, Karlsruhe, Germany) fixation. Flushed out BAL cells were centrifuged at 400g for 10 min at 4°C and used for flow cytometry.

### Lung tissue dissociation

The lung tissue was dissociated using the gentleMACS in combination with the tissue dissociation kit (Miltenyi Biotec, Bergisch Gladbach, Germany) according to the manufacturer’s protocol. Briefly, the lung was cut into the respective lobes and added into the dissociation tube with 2.4 mL of 1× buffer S, 100 μL of enzyme D, and 15 μL of enzyme A. Lung lobes were dissociated at 37°C for 30 min under rotation of the tube (program: 37C_m_LDK_1) in the gentleMACS (Miltenyi Biotec, Bergisch Gladbach, Germany). Isolated cells were collected by centrifugation (400g, 10 min at 4°C) and red blood cells were lysed for 5 min at RT, washed and resuspended in culture media (eBioscience, ThermoFisher Scientific, California, USA). Finally, cells were filtered through a 70 μm MACS SmartStrainer (Miltenyi Biotec, Bergisch Gladbach, Germany). Cell viability was assessed using DAPI staining after lung dissociation (S8C Fig).

### Purification of native proteins from the lung tissue

Lung tissue was placed into RIPA-buffer (eBioscience, ThermoFisher Scientific, California, USA) and lysed in a Precellys24 (VWR international GmbH, Langenfeld, Germany). Cells were incubated for 1 h at 4°C and centrifuged for 30 min at 16,000 g at 4°C. The supernatant was collected and adjusted to 100 μg/ml using Bradford protein quantification. Samples were diluted 1:100 in Bradford reagent (Sigma-Aldrich, MERCK, Darmstadt, Germany) and compared to a BSA standard.

### Detection of proteins from lysed lung tissue

10 μg lung protein extraction were used for the respective ELISA kits. CCL5, CCL11 (R &D Systems, Minnesota, USA), Amphiregulin (ThermoFisher Scientific, California, USA), major basic protein (Antikörper-Online, Aachen, Germany), MMP activity assay (Abcam, Cambridge, United Kingdom) and eosinophil peroxidase (Lifespan Biosciences, Washington, USA). ELISA measurements were performed according to the manufacturers’ protocols.

### Spleen dissociation

Spleens were isolated from the euthanized mice and dissociated using a syringe stamp and pressing the organ through a 70 μm MACS SmartStrainer (Miltenyi Biotec, Bergisch Gladbach, Germany). Splenocytes were collected by centrifugation at 400g at RT for 5 min and red blood cells were lysed (eBioscience,Thermofisher Scientific, California, USA). Cells were washed with RPMI1640 media (Gibco, ThermoFisher Scientific, California), counted and used for flow cytometry.

### Microfilariae quantification from lung

Lung and heart were isolated from the mice and placed into a glass petri dish. 20 G needles (B. Braun, Melsungen, Germany) were placed into the left and right atrium. An additional needle was placed into the right atrium and attached to a 10 ml syringe (B. Braun, Melsungen, Germany) containing PBS (Gibco, ThermoFisher Scientific, California, USA). The lungs were flushed with 10 ml PBS. The flow through was collected in a 15 min falcon tube and centrifuged for 10 min at 400g at RT. The supernatant was discarded and 1 ml of 1x red blood cell lysis buffer (ThermoFisher Scientific, California, USA) was added to the pellet. After 5 min at RT, the samples were centrifuged again at 400g for 10 min at RT. 950 μl of the supernatant was carefully removed. The pellet was resuspended in the remaining 50 μl and MF were counted via microscopy.

### Microfilariae quantification from peripheral blood

50 μl of blood was collected from mice in EDTA Tubes (Sarstedt AG & Co. KG, Nümbrecht, Germany) and transferred into 1.5 ml Eppendorf tubes (Eppendorf SE, Hamburg, Germany). 1 ml of red blood cell lysis (ThermoFisher Scientific, California, USA) was added and incubated for 5 min at RT. Following centrifugation at 400g for 10 min at RT, 1 ml of supernatant was removed, the pellet resuspended and all MF contained in the remaining 50 μl pellet were counted via microscopy.

### Flow cytometry

10^6^ cells were blocked in 1 μg/ml rat IgG/PBS (Sigma-Aldrich, MERCK, Darmstadt, Germany) for 1 h at 4°C. The blocking buffer was removed by centrifugation (400g, 8 min, 4°C) and the cell pellet was dissolved in 100 μl fixation buffer (BioLegend, California, USA). Cells were fixated at RT for 15 min and washed with PBS (Gibco, ThermoFisher Scientific, California, USA), centrifuged at 400g for 10 min at RT and stained in 20 μl surface marker antibody mix (S1 Table) for 30 min at 4°C. Stained cells were washed twice with PBS and centrifuged at 400g for 10 min at RT. For additional intracellular stainings, cells were washed twice with 100 μl permeabilization buffer (BioLegend, California, USA) and subsequently stained for 30 min at 4°C. Finally, cells were washed twice with PBS and measured using a CytoflexS flow cytometer (Beckman Coulter, California, USA). ILC2s were gated as CD45^+^, linage^-^, TCRb^-^, CD90.2^+^, ST2^+^, GATA3^+^, RELM-α positive macrophages as CD45^+^, CD206^+^, Siglec-F^+^, RELM-α^+^ and eosinophils as CD45^+^, CD11c^-^, Siglec-F^+^, CD11b^+^. Further, neutrophils were gated as CD45^+^, Ly6G^+^, alveolar macrophages as CD45^+^, CD206^+^, Siglec-F^+^. The gating strategy is shown in S9 Fig

### Enzyme linked immunosorbent assay

Invitrogen ELISA (IFNγ, IL-4, IL-5, IL-33) (Invitrogen, ThermoFisher Scientific, California, USA) were performed according to the manufacturer’s protocol from serum, bronchoalveolar lavage (BAL) or lung homogenates. Plates were coated with 1x coating buffer. 50 μl serum was diluted 1:2 in assay buffer, 100 μl of either BAL, serum or lung homogenates was transferred onto the plates alongside the respective standard samples and incubated for 2 h at RT and 200 rpm. Samples were blocked in assay diluent. Plates were washed and incubated with the detection antibody for 1 h at RT and 200 rpm. Plates were washed and horseradish peroxidase—conjugate was added for 30 min at RT and 200 rpm. Colorimetric detection was performed by adding 50 μl 3,3´5,5`-Tetramethylbenzimidin into each well. The reaction was stopped using 50 μl of 1 M H_2_SO_4_ (Carl Roth, Karlsruhe, Germany) to each well. Optical density of the plates was measured in a SpectraMax Molecular Devices LLC, California, USA) at 450 and 570 nm wave length. Concentrations were calculated using the Softmax Pro software (Molecular Devices LLC, California, USA).

### Detection of IgE

Parasite-specific serum IgE was detected by coating ELISA plates (Sigma-Aldrich, MERCK, Darmstadt, Germany) with 20 μg/ml female *L*. *sigmodontis* adult worm crude extract diluted in PBS (Gibco, ThermoFisher Scientific, California, USA) for 24 h at 4°C. The supernatant was discarded and plates were washed and blocked for 1 h with 5% BSA/PBS (MERCK, Darmstadt, Germany) at RT. Serum samples were diluted 1:50, 50 μl were added onto the plate and incubated for 2 h at RT. After the incubation, the samples were discarded and the plates were washed three times. The biotinylated anti-mouse-IgE antibody was diluted 1:200 (2μg/ml) in PBS, added to each well and incubated for 1 h at RT, at 200 rpm. Following a washing step, the plates were incubated with the conjugated horseradish peroxidase for 30 min at RT, shaking at 200 rpm. Colorimetric detection was performed by adding 50 μl 3,3´5,5`-Tetramethylbenzimidin into each well. The reaction was stopped using 100 μl of 1 M H_2_SO_4_ (Carl Roth, Karlsruhe, Germany) to each well. Optical density of the plates was measured in a SpectraMax (Molecular Devices LLC, California, USA) at 450 and 570 nm wave length.

### DNA quant

DNA quantification was performed as described previously [20]. In brief, the Invitrogen Quant-iT dsDNA Assay Kit, high sensitivity (ThermoFisher Scientific, California, USA), was used for DNA quantification of supernatant of stimulated bone marrow-derived eosinophils. Eosinophils were cultured in 96-well plates with medium containing murine IL-5 (20ng/ml PeproTech inc., ThermoFisher Scientific, California, USA). After 24 h, 2.5 U/well of the micrococcus exonuclease (PeproTech inc., ThermoFisher Scientific, California, USA) was added to all samples at 37°C for 15 min to disassociate DNA bound to the wells. The micrococcal nuclease reaction was stopped by adding 1 mM EDTA. Culture plates were subsequently centrifuged at 400g for 8 min at 4°C and the supernatant was removed and transferred to a new 96-well plate (Sigma-Aldrich, MERCK, Darmstadt, Germany). The quantification was done with 100 μl Quant-iT™ dsDNA HS buffer after a 1:200 dilution. The diluted DNA Quant-iT solution was added to 20 μl of the supernatant. For quantification, the provided standard ranging from 0–10 ng/ml was used. The absorption of sample and standard was measured using the Tecan infinity M200 (Tecan Group, Männedorf, Switzerland) at 485/525 nm.

### Lung histology

Mice were sacrificed and the lung lobes were inflated with 1 ml PBS (Gibco, ThermoFisher Scientific, California, USA) via the trachea using a 20G Vasofix intravenous vein catheter (B. Braun, Meisungen, Germany). To maintain the inflated status of the lung tissue, the trachea was closed with a yarn tied into a knot. The lung was removed together with the heart and placed into 2% methanol free formalin (ThermoFisher Scientific, California, USA) for 24 h at RT. The lung tissue was washed twice with PBS (Gibco, ThermoFisher Scientific, California, USA) and dehydrated by being submerged in ethanol baths of ascending concentration (50, 70, 80, 96, 100%) and finally 100% xylene for 30 min (STP-120 automated sample processor, Especialidades Médicas Myr, S.L., Tarragona, Spain). The tissue was embedded in paraffin (Merck, Darmstadt, Germany) at 56°C (modular tissue embedding center, Especialidades Médicas Myr, S.L., Tarragona, Spain). Hardened paraffin blocks were cut into 4 μm sections, paraffin was removed with alcohol baths, sections were stained with Mayer´s hematoxylin and eosin (Merck, Darmstadt, Germany) and mounted with entellan (Merck, Darmstadt, Germany). Stitched histology images were taken on the Axio-observer 5 (Zeiss, Oberkochen, Germany) using Zen V3.6 (Zen Blue edition V3.6, Zeiss, Oberkochen, Germany). Fifty 10x10 μm Random regions of interest were generated using KNIME (https://www.knime.com/ [59]) and lacunarity analysis was performed using the ImageJ [60] plugin FracLac (Karperien, A., FracLac for ImageJ; http://rsb.info.nih.gov/ij/plugins/fraclac/FLHelp/Introduction.htm). Protocol adapted from Chenery et al., 2019 [61].

### Statistical analysis

Statistical analysis was performed using Kruskal-Wallis followed by Dunn´s post-hoc test for comparison of multiple groups or Mann-Whitney-U test for comparison of two groups. A p value < 0.05 was considered as statistically significant. Statistical calculations were performed using GraphPad PRISM version 9 (GraphPad Software, San Diego, California USA, www.graphpad.com). Data is available via the Dryad database [62].

## Supporting information

S1 Fig (A) Number of microfilariae (MF) in 50 μl of peripheral blood 1 h after challenge injection of ELD WT and eosinophil deficient dblGATA mice as well as MF only challenged mice (MF). n = 10–18 animals per group. Data is shown as median with interquartile range. Statistical analysis was performed with Kruskal-Wallis followed by Dunn´s multiple comparison test. p values ≤ 0.05 are shown.
(TIF)

S2 Fig (A) Representative tissue sections of naïve, MF-challenged (MF), ELD, and dblGATA ELD mice stained with hematoxylin and eosin one day after MF challenge. 4 μm lung tissue sections.
(TIF)

S3 Fig (A) Splenocyte count of naïve, MF-challenged and ELD mice. (B) Spleen and (C) BAL eosinophil frequency (CD45+, Siglec-F+, CD11b+) of naïve, MF-challenged and ELD mice. Geometric mean fluorescence intensity (gMFI) of CD11b (D) and CD86 (E) of BAL eosinophils. Analyses were performed ten days after MF challenge. Data pooled from 1–2 independent experiments, n = 4–18, median with interquartile range. Kruskal-Wallis test followed by Dunn´s multiple comparison. p values ≤ 0.05 are shown.
(TIF)

S4 Fig Serum (A) IFNγ and (B) IL-5 levels of naïve, and 10 days after the final challenge of MF-challenged, ELD and dblGATA ELD mice. Data from one experiment, n = 5–8, median with interquartile range. Kruskal-Wallis test followed by Dunn´s multiple comparison. p values ≤ 0.05 are shown.
(TIF)

S5 Fig Representative photographs of murine lungs 10 days after intravenous challenge with viable MF. Lungs are shown for (A) a naïve mouse, (B) a mouse challenged with MF, (C) an ELD mouse and (D) an eosinophil deficient dblGATA mouse. Photos taken by BL.
(TIF)

S6 Fig (A) TSNE analysis of naïve, MF-challenged, ELD and HpARI2 treated ELD mice. Shown is one out of two representative experiments. (B) Eosinophil and neutrophils gated and (C) heat maps for CD54, CD11c, CD86, RELM-α, Ly6G, Siglec-F, CD206, CD11b, SSC-A, Ly6C. Analysis was performed with FlowJo.
(TIF)

S7 Fig Serum (A) total and (B) parasite-specific IgE of naïve and 10 days after the final challenge of MF-challenged, ELD and ELD mice treated with HpARI2. Absolute count of (C) neutrophils and (D) alveolar macrophages in ELD and dblGATA ELD mice. Data from 1–2 experiments, n = 6–8, median with interquartile range. Kruskal-Wallis test followed by Dunn´s multiple comparison or Mann-Whitney-U Test. p values ≤ 0.05 are shown.
(TIF)

S8 Fig (A) Purity and (B) viability of bone marrow-derived eosinophils after 24 h of *in vitro* culture. (C) Live/dead staining of lung cells after purification using different digestion methods. Data is pooled from two independent experiments, n = 6–12, mean + SEM. Kruskal-Wallis test followed by Dunn´s multiple comparison. p values ≤ 0.05 are shown.
(TIF)

S9 Fig Gating strategies for neutrophils, eosinophils, ILC2s, and alternatively activated macrophages. (TIF)

S1 Table Antibody clones and dilutions used for flow cytometry. (TIF)
