# Supplementary figures and images for: Repeated sensitization of mice with microfilariae of Litomosoides sigmodontis induces pulmonary eosinophilia in an IL-33-dependent manner
Source: PLoS Pathog. 2024 Mar 8;20(3):e1012071. doi: 10.1371/journal.ppat.1012071 (PMC10954174; doi:10.1371/journal.ppat.1012071)

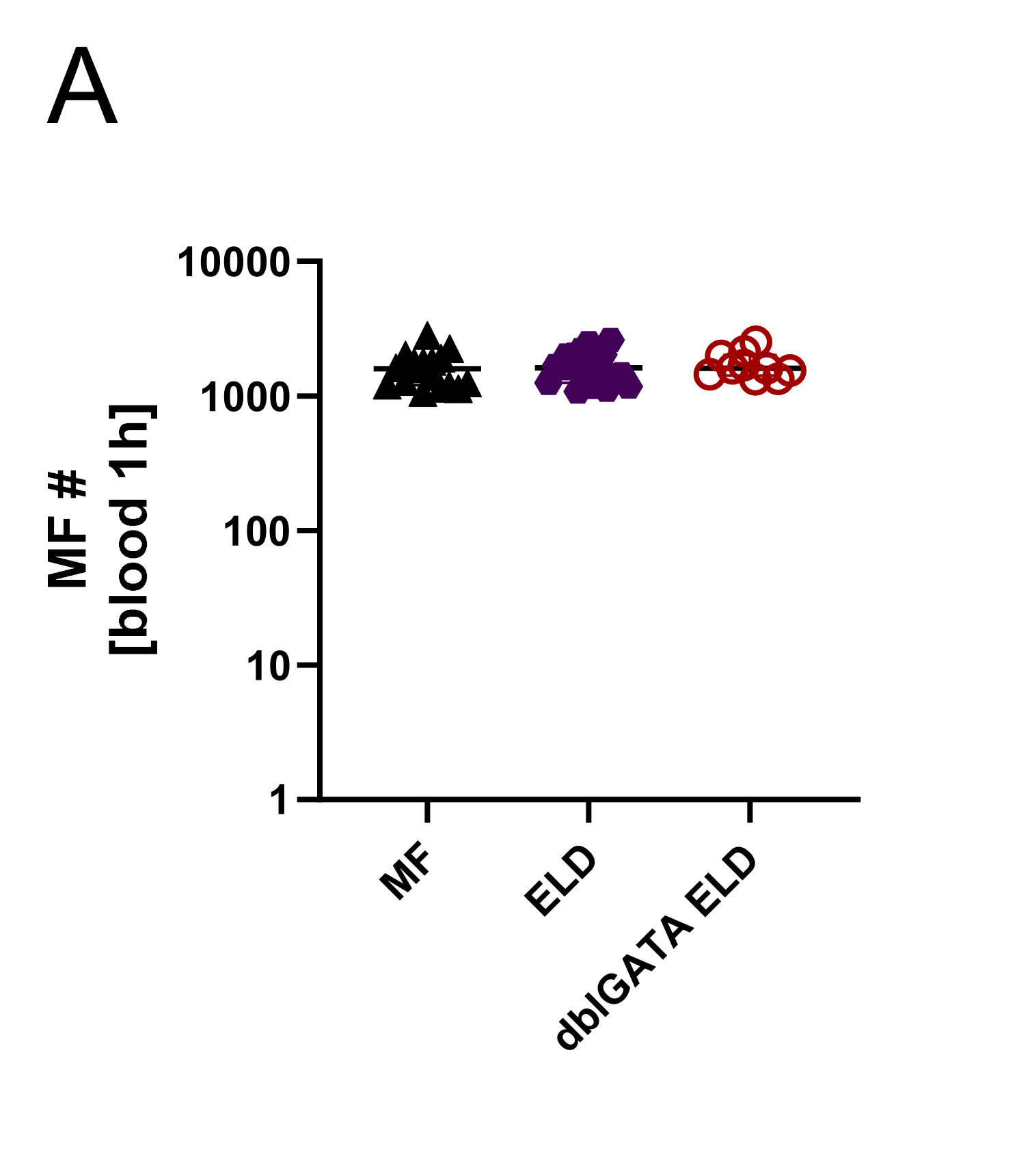

Supplement: S1 Fig — (A) Number of microfilariae (MF) in 50 μl of peripheral blood 1 h after challenge injection of ELD WT and eosinophil deficient dblGATA mice as well as MF only challenged mice (MF). n = 10–18 animals per group. Data is shown as median with interquartile range. Statistical analysis was performed with Kruskal-Wallis followed by Dunn´s multiple comparison test. p values ≤ 0.05 are shown. (TIF) [file ppat.1012071.s001.tif]

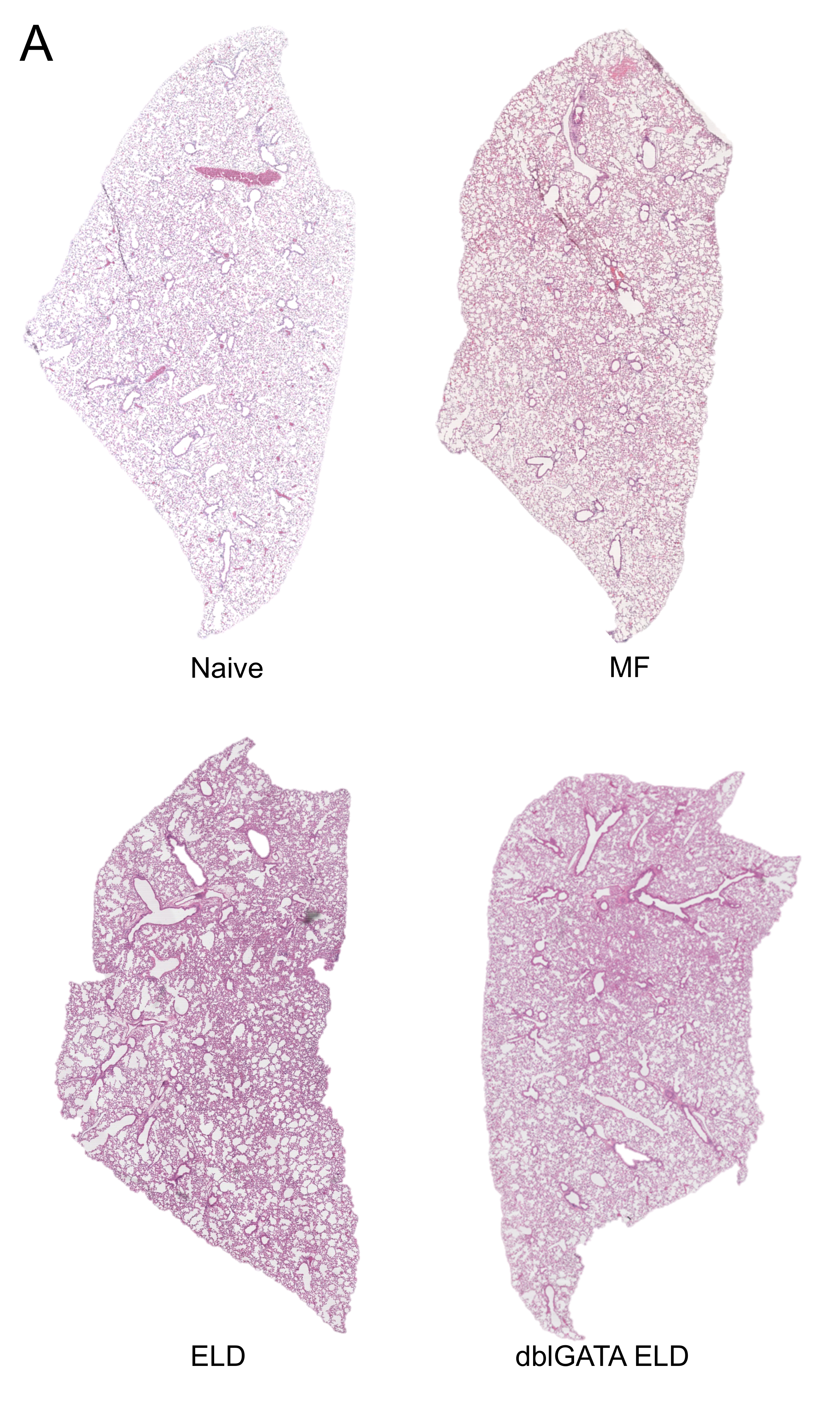

Supplement: S2 Fig — (A) Representative tissue sections of naïve, MF-challenged (MF), ELD, and dblGATA ELD mice stained with hematoxylin and eosin one day after MF challenge. 4 μm lung tissue sections. (TIF) [file ppat.1012071.s002.tif]

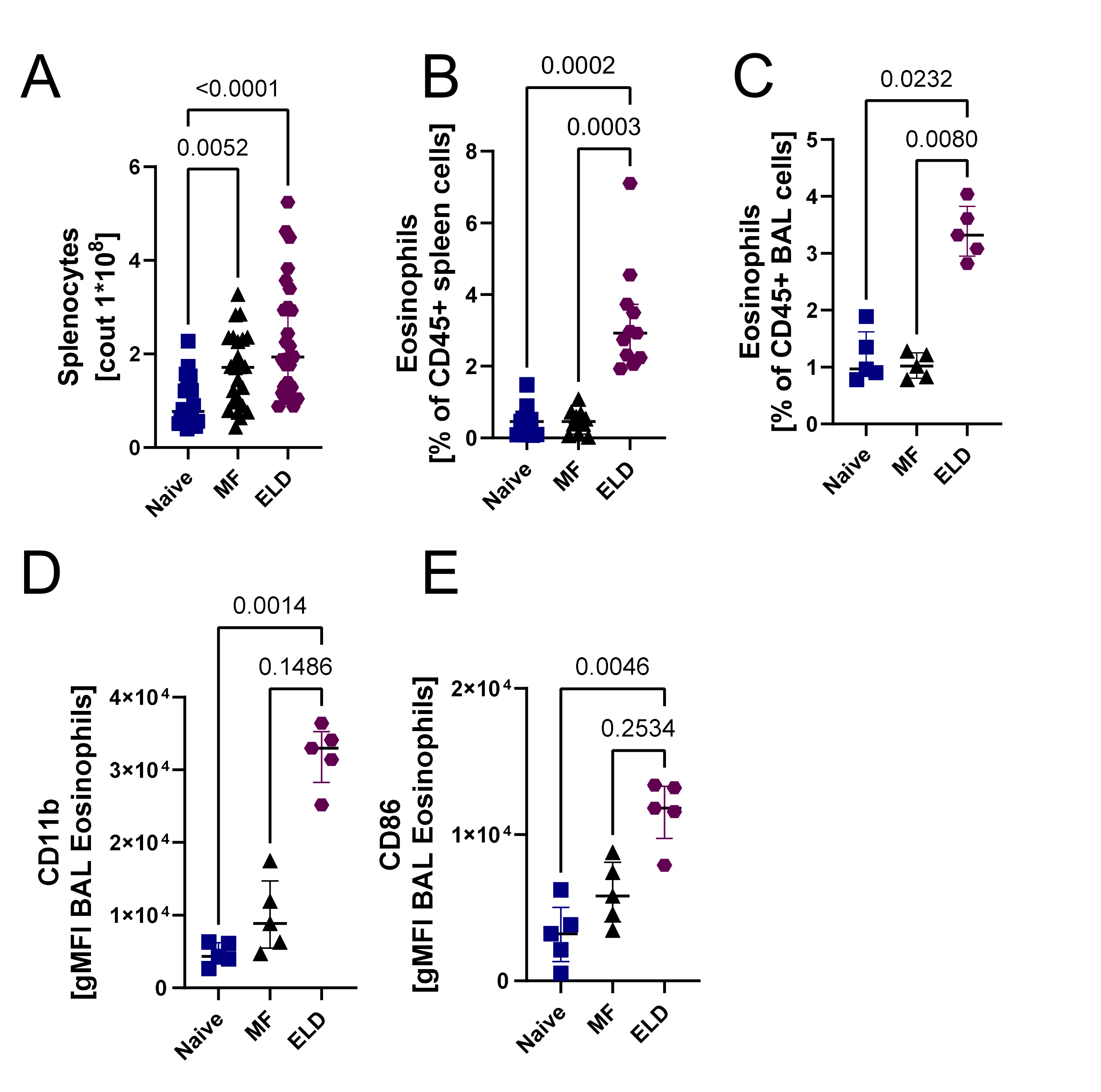

Supplement: S3 Fig — (A) Splenocyte count of naïve, MF-challenged and ELD mice. (B) Spleen and (C) BAL eosinophil frequency (CD45+, Siglec-F+, CD11b+) of naïve, MF-challenged and ELD mice. Geometric mean fluorescence intensity (gMFI) of CD11b (D) and CD86 (E) of BAL eosinophils. Analyses were performed ten days after MF challenge. Data pooled from 1–2 independent experiments, n = 4–18, median with interquartile range. Kruskal-Wallis test followed by Dunn´s multiple comparison. p values ≤ 0.05 are shown. (TIF) [file ppat.1012071.s003.tif]

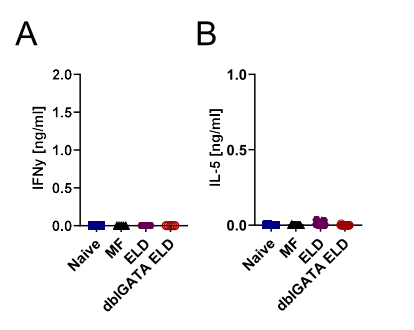

Supplement: S4 Fig — Serum (A) IFNγ and (B) IL-5 levels of naïve, and 10 days after the final challenge of MF-challenged, ELD and dblGATA ELD mice. Data from one experiment, n = 5–8, median with interquartile range. Kruskal-Wallis test followed by Dunn´s multiple comparison. p values ≤ 0.05 are shown. (TIF) [file ppat.1012071.s004.tif]

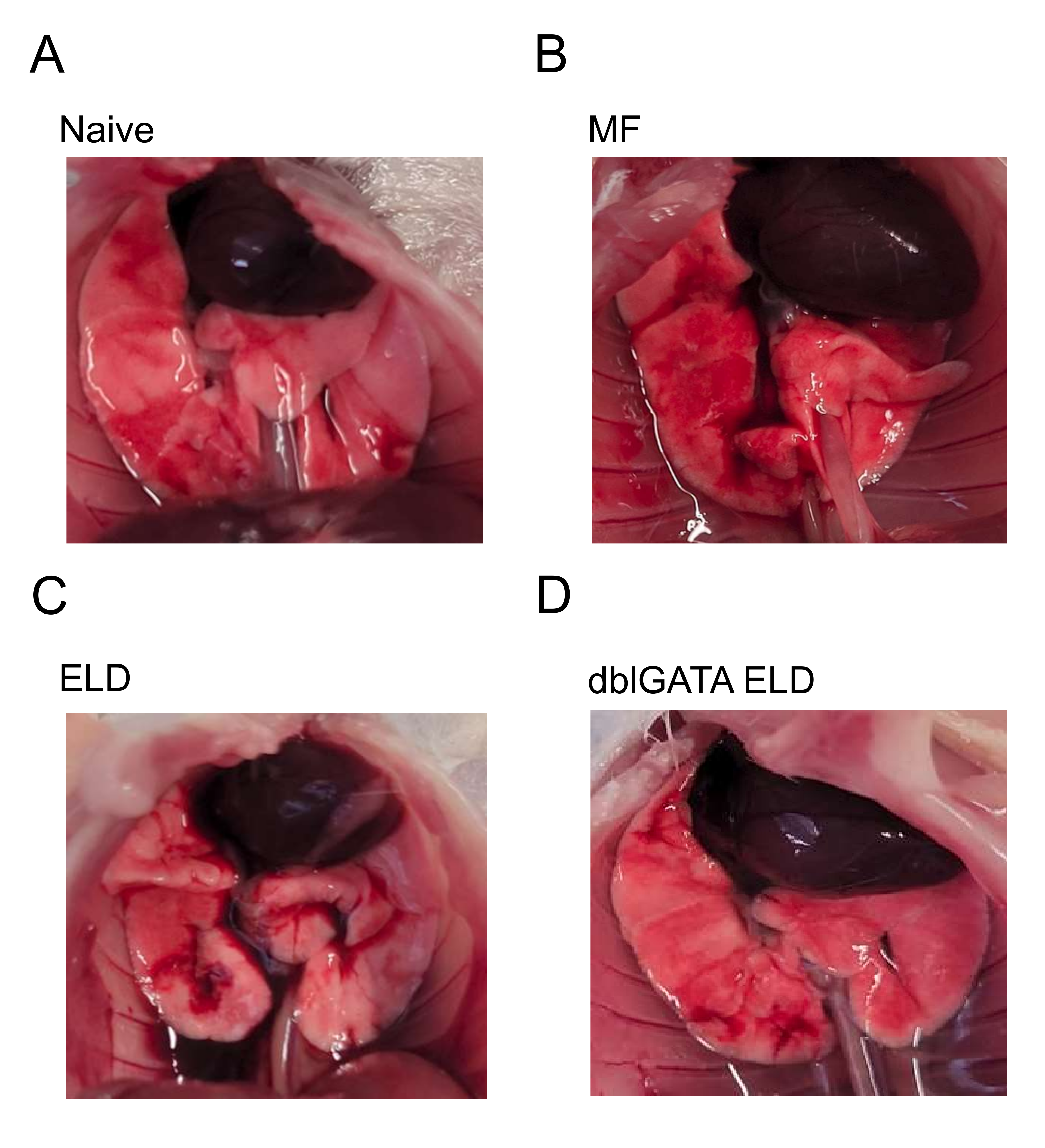

Supplement: S5 Fig — Lungs are shown for (A) a naïve mouse, (B) a mouse challenged with MF, (C) an ELD mouse and (D) an eosinophil deficient dblGATA mouse. Photos taken by BL. (TIF) [file ppat.1012071.s005.tif]

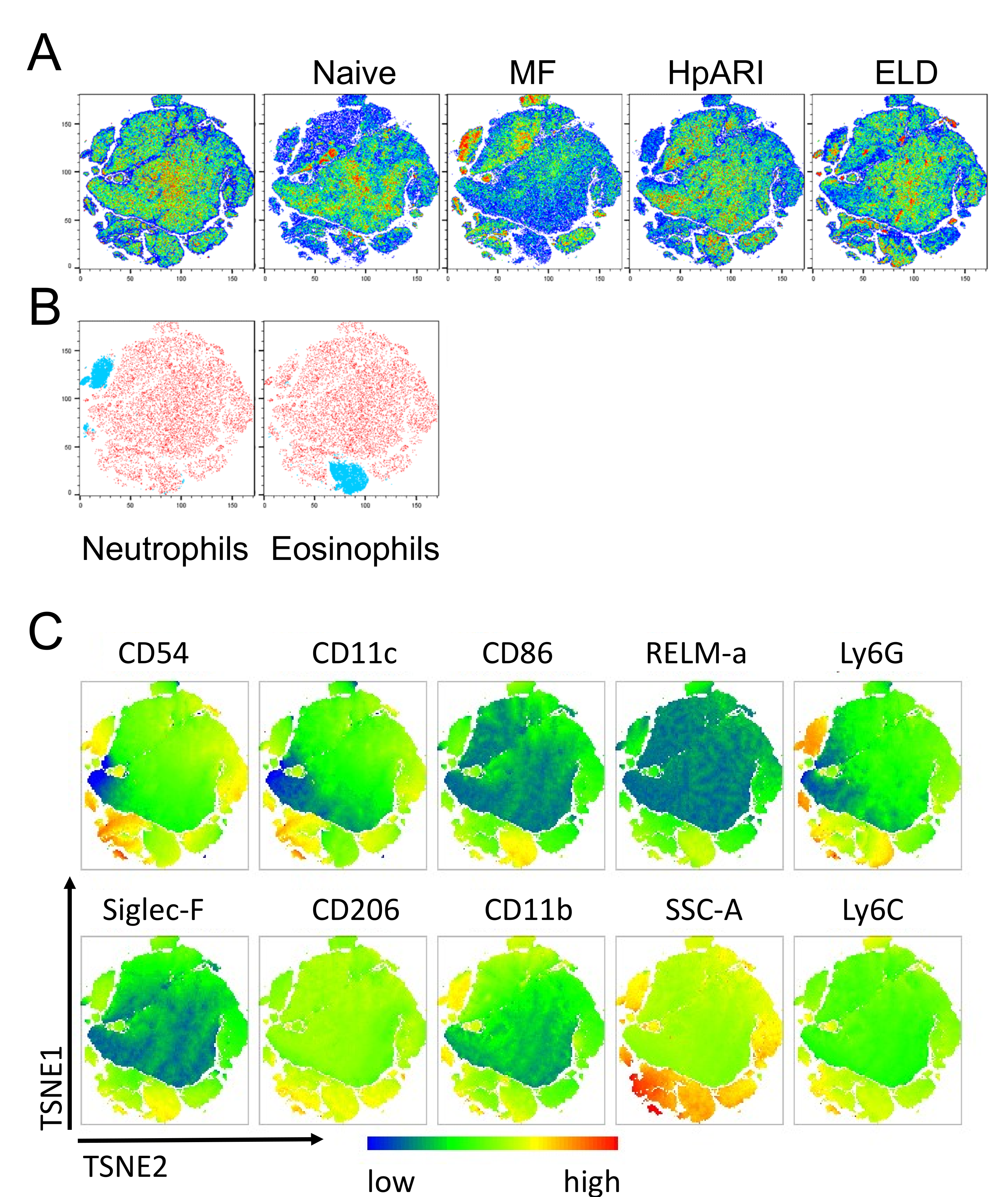

Supplement: S6 Fig — (A) TSNE analysis of naïve, MF-challenged, ELD and HpARI2 treated ELD mice. Shown is one out of two representative experiments. (B) Eosinophil and neutrophils gated and (C) heat maps for CD54, CD11c, CD86, RELM-α, Ly6G, Siglec-F, CD206, CD11b, SSC-A, Ly6C. Analysis was performed with FlowJo. (TIF) [file ppat.1012071.s006.tif]

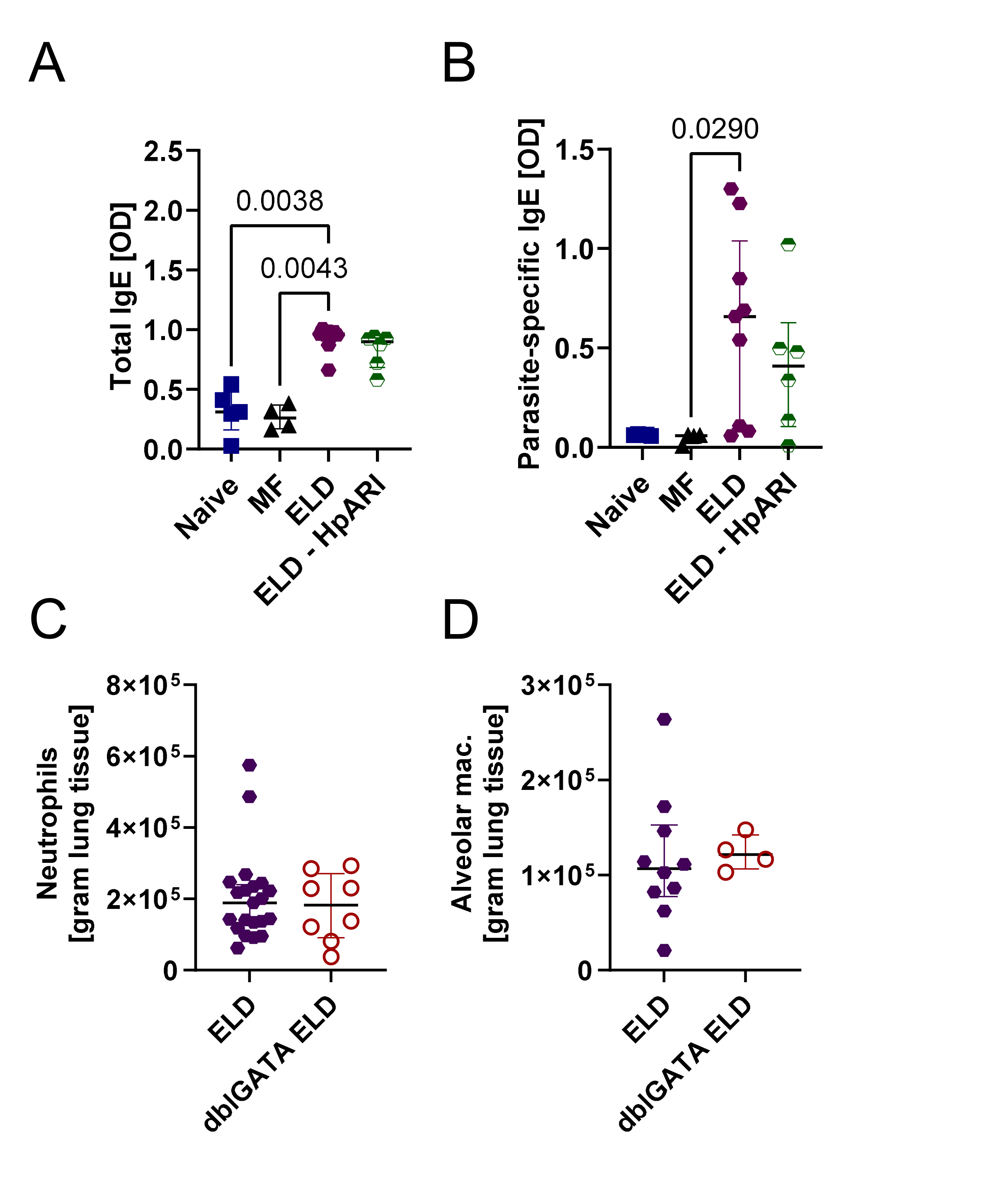

Supplement: S7 Fig — Serum (A) total and (B) parasite-specific IgE of naïve and 10 days after the final challenge of MF-challenged, ELD and ELD mice treated with HpARI2. Absolute count of (C) neutrophils and (D) alveolar macrophages in ELD and dblGATA ELD mice. Data from 1–2 experiments, n = 6–8, median with interquartile range. Kruskal-Wallis test followed by Dunn´s multiple comparison or Mann-Whitney-U Test. p values ≤ 0.05 are shown. (TIF) [file ppat.1012071.s007.tif]

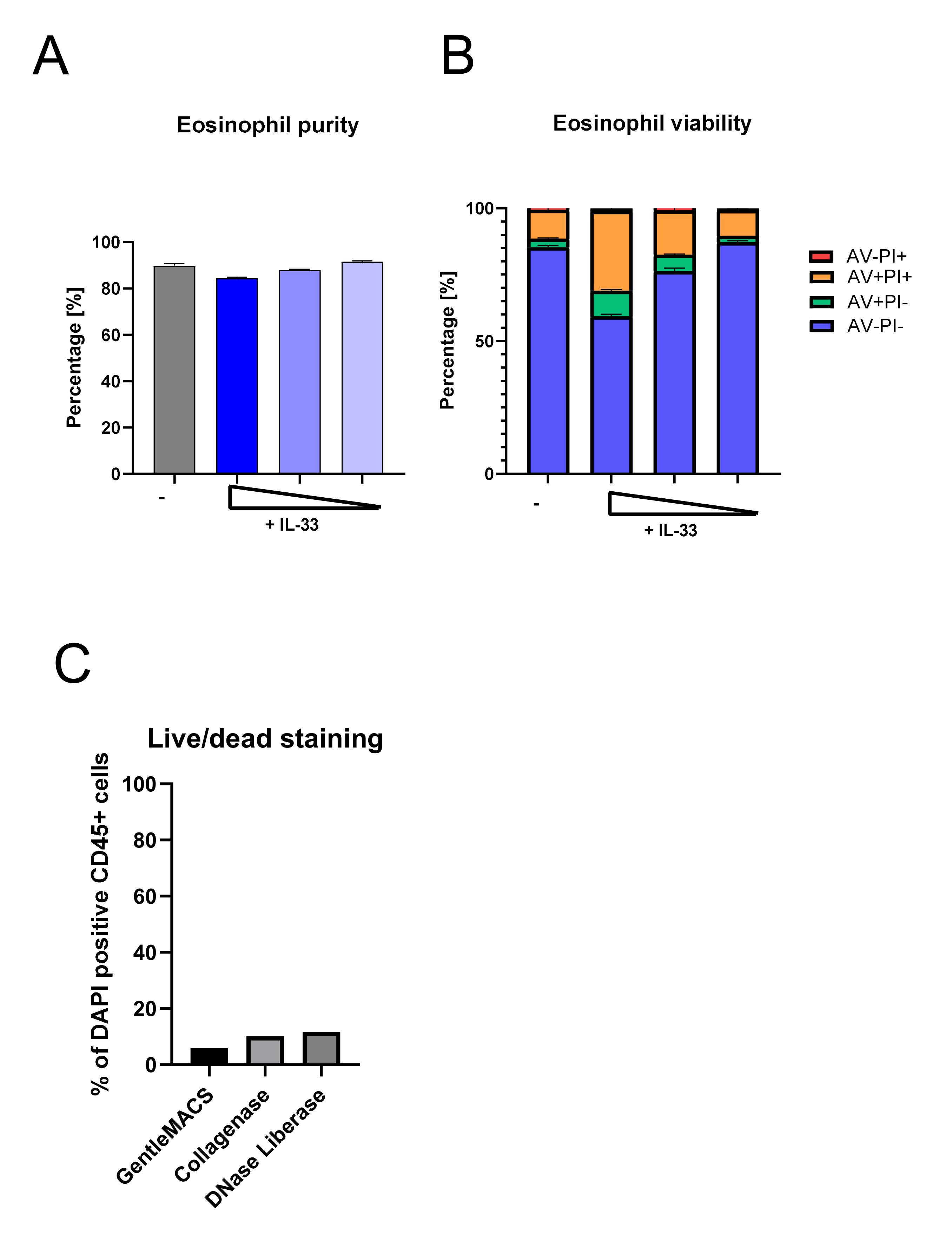

Supplement: S8 Fig — (A) Purity and (B) viability of bone marrow-derived eosinophils after 24 h of in vitro culture. (C) Live/dead staining of lung cells after purification using different digestion methods. Data is pooled from two independent experiments, n = 6–12, mean + SEM. Kruskal-Wallis test followed by Dunn´s multiple comparison. p values ≤ 0.05 are shown. (TIF) [file ppat.1012071.s008.tif]

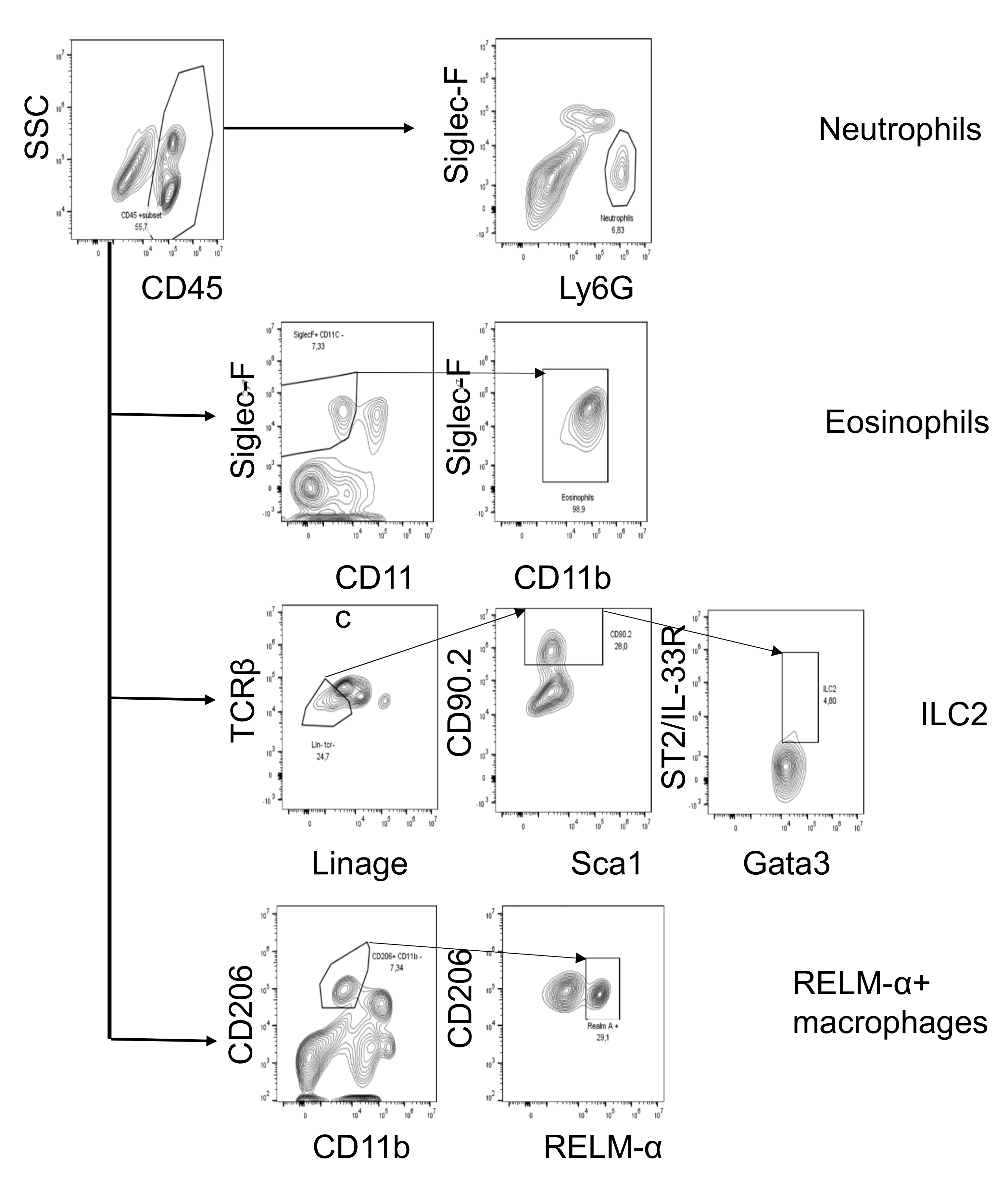

Supplement: S9 Fig — (TIF) [file ppat.1012071.s009.tif]

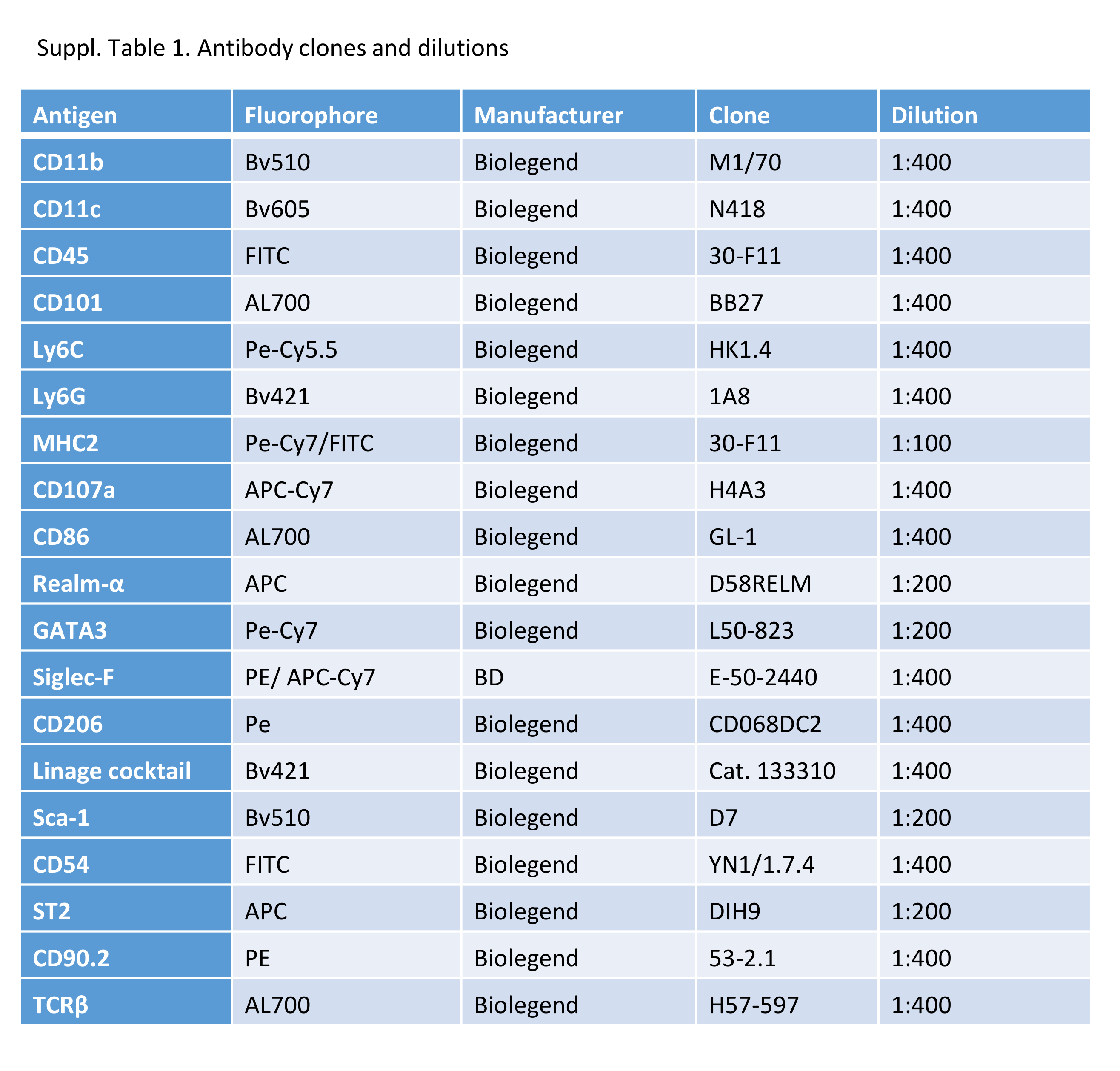

Supplement: S1 Table — (TIF) [file ppat.1012071.s010.tif]
